# Spirit of the English Periodicals, and Notices of English Medical Literature

**Published:** 1835-01-01

**Authors:** 


### Periscope;

OR,

### CIRCUMSPECTIVE REVIEW.

" Ore trahit quodcunque potest, atque addit acervo."

I.

### Spirit of the English Periodicals, and Notices of English Medical Literature.

Practical Hints on the Treatment of several Diseases. By Dr. J. Peacock, of Darlington.

This is an unpretending little volume, containing the veteran author's observations on the treatment of several ills to which flesh is heir. Dr. Peacock informs us that he is hastening to that bourne whence no traveller returns. We hope he has many days of bright sunshine before him on this side of that formidable pass. Be this as it may, he has laid his brethren under obligations for the present volume, which deals very little in speculation, but much in practice. The cases are detailed without any reference to order or nosological arrangement, being apparently copied verbatim from the case-book. The manuscript was submitted to the examination of Dr. Elliotson, who observed, that " the cases were highly interesting, and hoped the author would publish them, as his object was to do good, and acquire honorable reputation."—*Pref.*

I. A case of most extensive sloughing, not only of the tonsils, but of the whole of the fauces, was arrested in its destructive progress by the following mixture, which would, with difficulty, be borne by a sound throat.

℞. Confect. aromat.

Pulv. zingib. āā ʒj.

Carb. ammon. ʒj.

Tinct. opii, ʒij.

Aquæ menth. pip. ℥viij.

Misce et capiat coch. ij. mag. alternis horis.

II. A soldier quartered at Darlington, became jealous of his wife, and determined to starve himself. He lay in an open cow-shed, during cold weather, for five days, without food. When found, he was carried to a public house, and had brandy imprudently administered to him. This brought on violent inflammation in his legs, followed by gangrene. Spirituous applications were made to the legs, and a mixture, similar to the foregoing formula, was exhibited internally. At the end of four days " the skin, muscles, and fat were as cleanly dissected off as if done by Lawrence himself." Notwithstanding this he speedily recovered, and " was enabled to mount his new legs." We suspect that some error has crept into the text here. Had the *muscles* been destroyed, we doubt the probability of this soldier's ever mounting other than wooden legs afterwards.

Some other cases of sphacelus are related, where the good effects of opiates, stimulants, and nutritious diet were conspicuous.

III. *Diabetes.* Our author formed an opinion long ago, that diabetes originated in acidity of the stomach. He met with a military gentleman, aged 50, a good deal wasted with this complaint, all the usual symptoms being present. He prescribed ten leeches to the epigastrium, opened the bowels, and then exhibited five grains of carb. ferri, one grain of opium, three grains of pulv. aromat. ʒj. of creta preparata, the same of gum arabic, and three grains of pulv. jacobi veri, every four hours, day and night. He was told to use his own discretion as to food, but to drink only water. At the end of a fortnight he came to our author quite a new man. "From a withered old fellow he had become corky and facetious, and begged me to fix the day when we could have a bottle of wine together." Whether the doctor and patient cracked the bottle together, we are not informed; but the medicine was continued for a month longer, when he appeared perfectly recovered.

Our author has come to place great reliance on this plan, especially when the patients are not broken down with organic diseases. Two additional cases are detailed, and which support the author's assertions.

IV. *Dropsy*. Dr. P. has no specific for this complaint, which is, indeed, only the sequence of some other malady. In one case he was successful with Fowler's tincture of tobacco.

V. *Neuralgia Ophthalmica*. After a long prevalence of cold easterly winds at Darlington, in the year 1826, rheumatic complaints prevailed. In one case, a shepherd who had been much exposed, became affected with severe pain, irregularly intermittent, in the centre of his eye-balls. When the attack came on, it was generally attended by deep-seated pain in the head, and convulsions. The antiphlogistic plan had been tried, under the idea of cerebral inflammation; but Dr. P. adopted a stimulating treatment, consisting of tinct. guaiaci ammoniat. and opium, which succeeded, though with difficulty. A great number of similar cases afterwards occurred, and gave way to the same means.

VI. *Pain from Exhaustion*. Dr. P. is not quite certain whether or not he is right in the title of this chapter. Not being inclined to theorize, he leaves others to set the matter at rest. A gentleman, between forty and fifty years of age, came to Middleton, affected with a violent and deep-seated pain in his head, which, upon using any strong exertion, threw him into convulsions of the epileptic kind. He had been afflicted with moral ills of a distressing kind. His pulse was 86—eyes not red; but the pupils rather dilated—urine pale. He had undergone considerable depletion. After emptying the bowels, the following powder was given every six hours.

℞. Pulv. ipecac. compos. gr. vj.
——— aromat. gr. iij.
Sub. hydrarg.
Opii, āā gr. ss. M.

"This medicine released him from his sufferings very soon, so that, in a few days, he was able to go home."

"I was called in to consult upon the case of a carpenter, in Piersebridge, who had long suffered under an intense pain in the head, which had, with some reason, been supposed to proceed from occasional inflammation of the membranes of the brain.—The pupils of his eyes were rather contracted; his pulse from a hundred to a hundred and ten, when the pain was bad; and when it was upon him a sudden shake, or a false step, was very inconvenient.—The depletory practice was pushed to no purpose.—This case was a puzzle; but the compound ipecacuanha, with small doses of opium and calomel, restored him." 38.

VII. The three following cases are, (says Dr. P.) so incredible, that he is obliged to give the names, so that they may be authenticated, if doubted.—They will probably excite the risible muscles of some of our readers.

1. "H. Thompson, Esq. was brought up at Bowes, near Barnard-Castle, where he has a brother at present; he was of that class called yeomanry. But about middle life he engaged with two others in a very extensive commercial speculation, and happening to succeed, they were left immensely rich; and when this gentleman sent for me he had a most sumptuous establishment of carriages and servants. Mrs. Terry, of Darlington, had requested him to put himself under my care, and he took a house about half a mile from Darlington; but whilst his house was getting ready, it came into his head to consult a noted physician at Newcastle, from whence he returned without experiencing any relief.—This, he told me, was the thirtieth physician he had consulted since the commencement of the disease, and the fiftieth surgeon. His disease was constipation of the bowels; he had been six months and two weeks without any discharge that could be called a stool of any kind or consistence. When our patient was hungry, he swallowed any thing he took a fancy to; such as game, half-raw beef-steaks, rich soups, &c. &c. And about half an hour afterwards, he irritated his throat with two or three feathers, which made him disgorge what he had taken, and in a few hours he was fit for another meal. Sometimes he made a little urine, but very little and thick, and about an orange colour; and some such orange-coloured fluid as this came up with what he vomited: but it would have required the pencil of John Martin to have given any idea of the horrors which beset you when engaged in a tête à tête with this scarcely human being. The cuticle was nearly the colour of very old mahogany, and adhered so closely to the bone, that a careless person might have mistaken it for veneering, or a covering of the bone with a dark pigment.—Mr. T. was more than six feet high, with large bones, and so much emaciated, that I have proposed frequently, as a query, whether it was possible to scrape an ounce of flesh off his bones from any part of his body: he could not ride upon the softest carriage seat,—so that when he attempted exercise he rested upon his wife's arm, and could paddle about fifty yards in a day.—Their house, in Blackwell, was surrounded with a high wall, and frequently the poor villagers had to pay for the gratification of their curiosity, by peeping into the garden when he was using exercise; for it was not uncommon for the women and children to take fits when they got a glimpse of him,—indeed their screams were beyond description, and at length the gates had to be locked entirely. When you patted his sides with your fingers, you were conscious you were striking a large wallet of pebbles—he scarcely had any suffering which could be called pain.—I examined him very minutely, and among other questions I asked him if he was ever sick, and he said never in his life; at this I could not help breaking out into a laugh in his face, for which he gravely rebuked me for making his misfortune the source of merriment,—but I certainly never had so pleasant a laugh in my life, for it immediately struck me that this monster of a skeleton might be made well; and as it tickled my fancy, I laughed again and again; and I am not quite sure whether my patient did not send to his friend, Mrs. Terry, to ask if the physician she had sent him was quite SANE; however we commenced operations the very next day, and in less than two days he had discharged a full peck of excrement, which had a much nearer resemblance to a peck of black marbles than to any thing else; they were quite as hard, but rather larger, than marbles, and as black as jet, and very little of the natural fœtor. I had put together a very weak purging mixture of the common composition, two tablespoonfuls of which he was directed to take every four hours. —Now, the practised physician will see the occasion of my merriment when it broke out; and if I had not told him what I was about, he would have put five or six grains of antimon. tartar to ℥viij. What were the fifty surgeons and thirty physicians doing during the last six months that they could not hit upon a purgative as mild in its operation as manna is to an infant? for it really did not incommode him at all.—He had not pain nor griping. The only ALARM rested with the POTTERS, among whom, our grape shot made sad destruction. After this, Mr. T. got his flesh apace, and left Darlington in good health, as many here can bear evidence of."

"2. The following case was nearly as marvellous as the last. I was told that DIANA COLTMAN had come to see me from Winton, near Sigston, Northallerton; it was a warm day, and she had come extended upon a mattress on an open car, with very great difficulty; she looked as if she had been a good looking girl, but much emaciated; indeed the account she gave of herself was, that she had been confined in her bed upwards of three years, during which time she had never taken a mouthful of meat, not even bread.—When she attempted a small morsel, it threw a violent pain into her stomach, which was immediately felt in her head, accompanied sometimes with copious vomitings of blood, and loss of intellect.—At any time if her head was raised she lost her senses, and all power over her legs and arms; but her senses returned when she was laid horizontally. It was curious that ripe fruit never disagreed with her; she could take half an orange, a plum, or a cherry, with a high relish, and never threw them up—the same with gooseberries or grapes. The most striking feature was the loss of sense when her head was raised, and of muscular power in her limbs; when she came to me, her bowels were very slow, but by taking, during the day, ℈j. pilul. cœrul. hydrarg. and as much of the pilul. c. extr. colocynth and washing it down with a mild saline mixture, her bowels became temperate, and her mouth never affected; her urine was scarce till her bowels performed their office well, when we had no further difficulty; her tongue was dry and white, till she had got to take ℈j. of the blue-pill every day. She had frequently taken ʒss. a day, which never occasioned ptyalism; indeed I have remarked upon several occasions, that when a patient took large and repeated doses of mercury to act upon them, it was generally very successful when it did act. Whether this was a spinal case was never demonstrated—from its mode of cure I guessed it was. From the mystery in which the disease was enveloped, many unfavourable reports were circulated,—but I firmly believe without any foundation in truth; indeed the notice with which she had been honored by many respectable families in the neighbourhood, was a guarantee that nothing unfair had been practised upon their credulity. And the clergyman of the parish, a gentleman of considerable information, whose benevolence was very largely exercised in supplying the wants of this poor creature, had there been any attempt to counterfeit, would, long before she came to Darlington, have unveiled the impostor. By degrees she suffered her head to be raised a little several times a day, and the convulsions gradually subsided; but this was effected by a good neighbour, a very strong woman, who had the power of moving her in her arms with great facility, and lost no time during night and day to accustom her to a change of position. On her first arrival at Darlington, her stools were dark, but came gradually to their natural hue,—her diet, whilst here, was chiefly apples and pears, and when she attempted to make it more nutritive her sufferings gradually subsided. I think she was here about three months."

The third of these wonderful cases we must also give in the quaint and original language of the author.

3. "Mr. William Trufitt, a farmer at Huttonbonville, near Northallerton, had been sometime under the care of the faculty of that neighbourhood, in an obstinate diarrhœa; at last they sent for me; I forget how long he had been confined to his bed, but he was almost as shocking an object to look at as Mr. Thompson, with his black marbles. As soon as ever Mr. Trufitt eat any thing, he had a strong pressure to stool, which came away almost unchanged, and not in the least feculent; he had much thirst, and sometimes his stools were quite limpid, and as thin as gruel, and very little at a time; he really seemed as if he was made on purpose for drinking, although at other times a very sober man;—from being so very bony and thin, his skin was sloughing off from all parts of him. Astringent medicines by the mouth, as well as enemata, had been fully tried, but did not give ease; his pulse was forty—I was not to let him sink without an effort.—I recommended to his family to rub into his arms and thighs ʒss. ung. hydrarg. fort. during every six hours, night and day. I need not comment as I go along; the experienced physician will see what I am about;—

his diet was now lean broth without any vegetables. At the end of the sixth day there was no peculiar smell in his breath, nor any soreness or redness of the gums.—I was resolved, if possible, to have a determination to his upper works. They still rubbed on, and I gave my patient five drops of Fowler's SOLUTIO MINERALIS three times a day, in a spoonful of spring water.—On the fourth day of giving this medicine, the countenance was evidently bloated—but ptyalism had not ensued; but I nevertheless sang Io Pæans all the way home. It is only a man who has the interest and credit of his profession entirely at heart who can be aware of the pleasure I felt in returning home that evening. I had not miscalculated—he recovered gradually, but straight-forward, from that day. Ptyalism was a failure. We frequently meet with people who are proof against MERCURY,—but the enlargement of the head, and the eruption upon the skin, did the business equally well. In intricate cases, I have been frequently helped out by this revulsionary practice, and were it more studied in the hands of attentive practitioners, it might be brought to do much good."

VIII. In cases of obstinate constipation bordering on inflammation, Dr. P. avers that he "never failed to succeed when he was content with giving a quarter of a grain of calomel every quarter of an hour, particularly if he began with the calomel before the retchings came on." He justly observes that practitioners are often defeated in these complaints by giving too much medicine by the mouth, and thus increasing the irritability of the stomach. In cholera, our author and his partner, Dr. Macfarlane, tried the plan of giving a grain of calomel every ten minutes, washing it down with a little cold water. They did not lose a patient.

IX. *Pain in the Stomach, with Indigestion.* Dr. P. is a stanch advocate of the Broussaian doctrine of dyspepsia —namely, that it consists of chronic gastritis, and lauds the exhibition of "mucilaginous drinks acidulated with lemon-juice, and debarring his patients from eating animal food, &c." We suspect that our veteran confrere of Darlington has not suffered much from indigestion, or he would not have been so warm in his praises of acid slops in that complaint. The notorious fact that dyspeptics can (nine out of ten) digest animal food much easier than vegetable, is a proof that the disease is far more nearly allied to gastralgia than to gastritis.

X. *Headaches.* Dr. P. must surely regret his advance on Time's list, since he must have been one of the most successful practitioners that ever lived. Most physicians become sceptical as to the great efficacy of medicine, as they get old; but Dr. Peacock is just the reverse. His remedies are infallible, and consequently his success complete.

"We frequently meet with people who have most violent and distracting head-aches from various and opposite causes, yet always difficult of relief.—The cases are so one like another, and the cure accomplished by such simple means, it is hardly worth while running into the detail of cases—although my memory would furnish me with ten or twenty at a call. But let those who are subject to such dreadful pangs take the following medicine as directed, and I shall be much disappointed if a few minutes does not convince the sufferer of its efficacy.

℞. Bac. Capsici. ℈ij.
Gum Arabic, gr. x.
Syr. Alb. q. s. ft. Pil. xx. quar. sum. iv. 2da. quaq. hora dolore urgenti.

It is useful to wash them down with a little cold water—but there is seldom occasion to take more than one dose as they take away the pain like magic."

XI. For sciatica and tic douloureux, which our author considers as twin-sisters, his sheet-anchor is the seeds of stramonium. The following is the formula he employs.

℞. Bac. Capsici, ʒj.

Calomel, gr. x.

Sem. Stramonii, ʒij.

Muc. G. Arab. q. s. Ut ft. pil. lx. quarum sumat. 1 quarta quaque hora. He also administers a couple of grains of opium occasionally during the continuance of the pain. Five drops of Fowler's solution are also given twice a day.

We have now extracted the substance from Dr. Peacock's book, and given it a greater extent of publicity than it would be likely to attain as a monograph—or rather a polygraph. We are not much disposed to censure mere manner or style in a medical book; but we think Dr. Elliotson would have done the author of the work which we have reviewed a favour, if he had smoothed some parts of the language employed in it. Dr. P. is evidently one of the old school; and we have no right to expect the introduction of modern pathology in a brochure, which merely aims at conveying some practical hints to the author's less experienced brethren.

---

### J. LATHAM, ESQ. ON UTERINE HÆMORRHAGE.

This subject has occupied much space, of late, in the medical journals. In our Liverpool contemporary of June last, there are two papers on uterine hæmorrhage in the same Number—one by Mr. Banner, the other by Mr. Latham. The latter gentleman writes from an experience of more than 30 years, and his observations are deserving of notice. He remarks that, if a patient has suffered from hæmorrhage in a former confinement, he would rupture the membranes as soon as practicable, with the view of exciting the uterus to early contraction. When the pains are violent, threatening hasty delivery, he endeavours to retard somewhat the expulsion of the child's head by moderate pressure on the perinæum. The body of the child, also, he leaves to the natural expulsion of the uterus. He strongly advocates a band round the abdomen with several folds of napkin over the region of the uterus. A careful and early examination of the uterus should be made, and its contraction hastened, by extending the hands over the uterine region, and firmly and evenly grasping it, the patient lying on her back. "Time and experience (says he) have convinced me of the advantage of these means in particular, when persevered in, over all others."

---

### THE TRIUMPH OF TRUTH AND GOOD SENSE, &c.*

The design of this little brochure is undoubtedly good, and the execution may be respectable; but the result must necessarily prove a decided failure. "The triumph of truth!" Dr. De Mey must be a greenhorn. In our younger days—"when the heart promised what the fancy drew"—we dreamt of such triumphs, and frequently aspirated the adage of the sage —"omnia vincit veritas." But we have long ceased to entertain such Utopian notions—as far, at least, as quackery is concerned The very motto of the pamphlet before us—the text of the author himself—might have taught him the inutility of his sermon.

"The world is nat'rally adverse
To all the truth it sees or hears;—
But swallows nonsense and a lie,
With greediness and gluttony."

How, then, can Dr. De Mey expect that the world will swallow his "Triumph of Truth," when it has so many of Morison's pills and other nostrums to swallow? He might just as well "whistle jigs to a mile-stone," under the hope that that steady personage would dance a minuet to his music, as preach to the world against the dangers of quackery, the excoriations of Long, and the drenchings of Morison and Co. Besides, the public might very naturally turn round on the worthy Doctor, and ask him what he thought of such taking titles as "Consumption curable," and many other *regular* quackeries! We think that medical men had

* By Dr. De Mey. Octavo, sewed, pp. 30. 1834.

better leave admonitions of this kind to judges, magistrates, coroners, and non-professional writers, since harangues against charlatanism are always read with distrust, when they emanate from the faculty. The evil must cure itself, by the punishment that pursues folly, in physic as well as in every thing else. Solomon has said, and Dr. De Mey has quoted the sentiment—that "bread of deceit is sweet to man; but afterwards his mouth shall be filled with gravel." If, therefore, the various classes of society, from the peer to the peasant, prefer gamboge to other medicines for the cure of all diseases, let them have their choice, in God's name! In no other country is there a greater redundancy of population than in this —in no land is quackery more effectual, as a preventive check to this redundancy than in happy Old England. Why, then, attempt to cripple its beneficial influence by admonitions, which are seldom read by the public, and generally distrusted, if read. So deeply rooted in the human mind is the love of quackery, that even the frightful exhibitions of Long's and Morison's handyworks produce but a momentary sensation. In fine, if quackery be an evil, medical admonitions, ex cathedra, will never mend it. We, therefore, advise our brethren to be silent on the subject, except when their evidence is solicited in courts of justice.

---

A Treatise on Gout. By David Leese, M.D.

Dr. Leese has been very moderate. He has only given us a small brochure of 47 pages on the intricate subject of gout. Yet he might have condensed his treatise into 47 lines, without much loss to his readers. The author became affected with gout 31 years ago, and treated himself, with indifferent success, till the year 1818, when his attention was attracted to Sir Everard Home's paper on colchicum. He had recourse to this remedy, in small doses, combined with vinum ipecac. and has experienced the best effects from the said combination, aided by proper regimen. This is the sum and substance of the pamphlet. We subjoin an extract respecting diet, which is not uninstructive.

"It may be proper in this place to remark, contemplating the disease, as I do, a variety of inflammation, that during the early stage of an attack, when leeching may be found necessary, the diet of the patient should be in accordance; consequently, a more abstemious system should be resorted to than he is accustomed to in health, or when the violence of the attack has been subdued. I shall not say much on the subject of diet, the disease does not often attack persons till they arrive at years of maturity, when every one's common sense should teach him to avoid what he has found to disagree with him; keeping in mind that intemperance and excess are fruitful sources of disease, especially of Gout, and as it is not in accordance with my opinion to treat it as an humoral disease, there is less occasion to go into minutiæ on this head. Nevertheless, it is necessary to give some general advice on that point. Much must depend on habits and station in life:—and, first, it may be observed, that excess in quantity, and in variety of wine or viands, is *nearly as much** to be deprecated as quality; to blend a variety of heterogeneous articles in the stomach is doing the utmost to produce a fermentative process there; engendering thereby flatulence, dyspepsia, and, consequently, imperfect chylification. Who has not felt or found the acid eructations consequent on such a proceeding? Abstinence is not necessary for the gouty, but simplicity in diet is essential. At the principal meal, or dinner, which should not be taken later than four o'clock, there is no objection to mutton, beef (not salted), lamb, poultry, game, or venison, but not with high sauce; or, fish *in season*, and alternately; but let not the arthritic partake of more than two of these at one

---

* He might have said "much more."—Eds.

repast, avoiding generally, pastry, and what are called made-dishes; batter or bread-pudding may be added to the two articles *selected for the day.* Spirits in all their various forms, and delusive names, I hold utterly objectionable, and malt liquor should be avoided, considering it much better to drink water, or toast and water during dinner, even to the admission of a glass more wine than might otherwise be allowable. If soup be taken, it should be of the simplest kind; for instance, gravy with vermicelli. Brown bread made of flour, from which only the coarsest bran has been taken (but none of the farinaceous parts), may, with great advantage, be substituted for the bakers' bread of London; if this cannot be obtained, biscuit may be taken. Wine is a very important consideration connected with the diet of that class of persons subject to Gout. The quantity should depend much on previous habits; but, I am of opinion, as a medium rule, a pint of sound wine may be taken with impunity during and after dinner, when free from an attack; and the first rule as to choice, I think, is, that which has been found to agree best, avoiding the delusion, though, of considering *that* the *best,* which has been found most apt to fix a wandering, or *direct* a misplaced Gout, to some part more agreeable to the fancy —and I am of opinion that Madeira wine, from some latent acid or other quality, has obtained so much favour, because it is *most productive of Gout:* if the digestive powers of the stomach be good, I know of no wine better than sound old Port, that has deposited its tartar; should this not agree or not please, the next to be preferred is sound old Sherry. I have drank some Hermitage, to which I think no objection could be made. Claret may be taken, but should be carefully chosen, none but the very best being admissible. Two or three glasses of Sauterne wine may be drank (drunk): Hock and Cyder should be avoided.

With these few and simple remedies I have named, may every case of Gout be combated and subdued, as certainly and safely as any other severe disease, and all the deformity and decrepitude we too often witness prevented."

Dr. Leese has amused himself (probably during some comfortable immunity from gout) with a laugh at the absurd speculations which have been hazarded by eminent physicians, ancient and modern, respecting the nature and treatment of this painful malady. We have been too poor and too industrious to be honoured with visits from this aristocratic guest—therefore we cannot lay claim to so much personal experience as Dr. Leese is possessed of. We have seen a little of the complaint, however; and we cannot by any means agree with our author, that gout differs in nothing from common inflammation, except from the nature of the parts which it attacks. Why does gout so generally affect certain parts? Why did Dr. Leese so regularly have attacks of inflammation in his *great toe,* after indulging in East India Madeira? This looks something like a specific inflammation. How did it happen that the worthy Doctor's nose did not sometimes turn red, instead of his great toe?

---

### Hemiplegia in Pregnancy.

There are very few cases on record, where a female has suffered apoplexy and hemiplegia in the last stage of pregnancy, and come through delivery with ease and safety. A case of this kind is recorded by Dr. O. Roberts, in the Liverpool Medical Journal for June last. The female, aged 40, was seized with apoplexy in the middle of the ninth month of pregnancy. She was copiously bled, and when she came to her senses, one side was completely paralysed. Nevertheless, labour took place, and both child and placenta were expelled before the accoucheur could arrive. The uterus contracted well, and no flooding occurred. A case nearly similar is related by Dr. Kellie, of Leith, in Cheyne's work on apoplexy. The woman was in the last month of pregnancy, and had a regular apoplectic attack, followed by complete paralysis of the right side. She was safely and easily delivered of a living child. She died of the apoplectic attack, however, a day or two afterwards. These two cases are deserving of record, as marks to guide the prognosis in similar emergencies.

---

### Carditis—Cephalitis?

In the fifth Number of the Quarterly Medical Review, Dr. Stroud has narrated, with great minuteness, a case of the above nature. The patient was a married woman, aged 31 years, the mother of four children, and then suckling, though with difficulty. She had been labouring for a fortnight previously under febrile symptoms, with pain in the back of the head, at the pit of the stomach, and in the loins and limbs. She now has cough, sleeps little, and is sometimes delirious. The pulse is 130, and weak. The tongue white, with red edges—thirst—anorexia—urine natural—bowels confined. The cause of these phenomena is attributed to fatigue, anxiety of mind, and exposure to cold air. Leeches to the temples—salines—sudorifics. This was on the 15th Nov. 1831. 20th. Not much change. The head to be shaved, and the infant weaned. The pulse is nearly 140, and weak—troublesome cough, and hurried respiration. *Soothing medicine.* 22d. Pain in the back of the head severe, with some intolerance of light—face flushed—pupils contracted—sleep interrupted—delirium occasional. Cupping—mercurial frictions—salines. 23d. Pulse 120 to 140, and very weak—tinnitus aurium—cough troublesome, with "hurried breathing, frothy expectoration, and pain in the middle of the chest." Venesection to eight ounces—blister to the chest—salines—mercurial frictions. 25th. Bore the bleeding well—blood nearly natural—gums slightly affected—pulse 130, and feeble. 27th. Cough dry—breathing hurried—delirium—pulse very frequent and feeble—"respiratory murmur, now for the first time examined, is sufficiently audible." Venesec. ad ℥viij., salines, aperients, digitalis. 29th. Relieved by the bleeding—blood nearly natural. "The action of the heart, examined this day for the first time, was found to be very strong, although the pulse at the wrist was extremely small and weak." Venesection to 12 or 16 ounces—leeches to the præcordial region, &c. Dec. 3d. The bleeding and leeches gave relief. "The patient is considerably better." Pulse 112, hard dry cough. 5th. Patient rather worse—pulse 120. V.S. ad ℥viij. vel ℥xij. 7th. A miliary eruption has appeared on the head and neck, accompanied by profuse perspiration. The heart sometimes palpitates; but the breathing is tranquil—pulse 120, and weak. 18th. Pulse 140, and excessively weak. On the 28th, we find that a previous bleeding had given great relief, and that the pulse had fallen to 98, the action of the heart being tranquil, and the breathing easy.

We need not pursue the narrative any longer. She menstruated on the 8th of January; and we find that, in the middle of February, the pulse was still 120 and weak, in the sitting posture and after walking. She afterwards slowly recovered, retaining for some time a disposition to irregularity of the heart's action. She became pregnant—was safely delivered, but unable to suckle well her child. In February, 1833, she had a slight return of the cardiac affection, from which she recovered by antiphlogistic treatment.

*Remarks.* We were a little surprised, on perusing the above case, to see that auscultation was not employed till 12 days after Dr. Stroud had commenced his attendance—and, even then, the action of the heart was not examined. Two days afterwards, when that organ was examined, we venture to say that Dr. Stroud was surprised to find it in a state exactly the reverse of that indicated by the pulse. The depletive measures were then employed with a bolder hand, and we find a corresponding relief obtained. The case is very valuable, as exemplifying the exceeding fallacy of the pulse. Very few would have ventured on venesection at all, if guided by the state of the pulse in the foregoing case ; yet we have not the smallest doubt that carditis was going on from the beginning. We are inclined to think that the affection of the head was secondary, or owing to the rapid circulation rather than to actual inflammation. Nothing is more common than these sensorial disturbances, when the central organ of the circulation is in a state of inflammation, or even excitement. We have no fault to find with Dr. Stroud's pathology or therapeutics; but we are much deceived if the above case does not put him more on his guard, in similar cases, for the future, and induce him to compare the action of the heart itself with that of the arteries, whenever there is any doubt as to the state and condition of the circulation. We advise all practitioners to examine the state of the heart and lungs in *all* cases—were it only for the sake of becoming intimately acquainted with the *normal*, as well as abnormal functions of the thoracic organs.

---

### ENDEMIC DYSENTERY.

The Edinburgh Charity Workhouse was visited with a severe dysentery, in the years 1832 and 3, of which Dr. J. Smith has given an account in a recent Number of our Edinburgh contemporary. The house stands in the healthiest part of the town, but the inmates are generally infirm, especially on entering the asylum, though they gain flesh and retain good health while there. The diet is chiefly vegetable and farinaceous, with a small proportion of animal food. During Summer and Autumn, they are usually affected with bowel-complaints, "as diarrhœa, dysentery, and English cholera." In the Winter and Spring of 1832, when the epidemic cholera was at its height in the Lunatic Asylum, 150 yards distant from the workhouse, the inhabitants of the latter escaped—partly, Dr. Smith thinks, from the rigid quarantine established there ! Nevertheless, in July of the same year, dysentery broke through the rigid barrier, and scourged the paupers. That this dysentery was a grade of the epidemic cholera which prevailed in the less healthy parts of the town, is as clear as the sun at noon day. The following description will satisfy the most sceptical.

"The attack generally commenced with vomiting and purging, pain of the abdomen, and a considerable degree of fever, the pulse in many cases being frequent and sharp, and the skin dry and hot. In others, however, a degree of collapse followed the vomiting and purging, the pulse was small and feeble, the surface cold or covered with clammy perspiration, and the features sunk. In some cases diarrhœa went on for several days, when *tormina* and *tenesmus* succeeded. The matter vomited was generally the ingesta or mucus. It was seldom or never bilious. The pain of abdomen varied very much both in degree and in situation, although it was very generally referred to the umbilical and hypogastric regions.

The epigastric region was often the seat of it, and then there was great irritability of stomach, and vomiting, and a constant burning sensation. In some, the abdominal tenderness was very trifling ; but this by no means indicated a mild form of the disease, for the patients to whom this happened, feeling no pain, often allowed the disease to go on till it was in its worst stage. The state of the tongue was various ; at the commencement, when fever was present, it was dry and furred. It was often coated in the centre, but moist and red at the edges. This might be considered the most frequent condition of the tongue. In the most aggravated cases it was dry, parched, and red; and in some, when the other symptoms denoted a severe form of the disease, it was almost natural. Thirst was generally an urgent symptom, but more particularly in the advanced stage of the disease, when there was great gastric pain and irritation. In such cases there was a constant desire for cold water to relieve the burning sensation in the stomach.

The sound of the voice was in many cases peculiar ; it was low and whispering, particularly when there were symptoms of collapse. The respiration was not affected in such a way as to be remarked as a characteristic symptom. Tenesmus was present in every case, and in the advanced stages the distress attending it was truly harassing. The desire to go to stool was constant, from the feeling that there was always something in the rectum to be evacuated, and the patient, if allowed, would never have ceased straining.

The appearance of the dejections was very various, but always without bile."

To us it appears clear, that the above disease was owing to the general epidemic influence then prevailing, but modified by the insulated locality, diet, and other circumstances of the institution.

On dissection, the large intestines were found thickened, ulcerated, and otherwise organically affected. The submucous tissue was highly injected. These changes were far from being the cause of the dysentery—they were most undoubtedly the *effects*. They aggravated the disease, and were the chief cause of the fatal termination; but they were not the *cause* of the dysentery. In many fatal cases of typhus, we find ulceration of the bowels; but it is an erroneous opinion, that these ulcers constitute the essential pathology of typhus.

*Treatment*. Our author having witnessed the good effects of large doses of calomel in Asiatic Cholera, and having failed in his treatment of the dysentery by the more common means, had recourse to scruple doses of the submuriate.

"It occurred to me, that between the symptoms of cholera and the endemic I had to contend with, there was considerable resemblance. Both diseases came on in the same way, by vomiting and purging. What was ejected, although not the same in both, yet agreed in this particular circumstance, that it was without bile. There was a burning sensation at the pit of the stomach, along with a desire for cold water in both. The *post-mortem* appearances exhibited this similarity, that the gall-bladder in both diseases was found invariably distended with bile."

The result proved that he was right in his conjectures respecting the affinity of the two diseases. He exhibited calomel in scruple doses, and "the immediate results of the experiment went far beyond my most sanguine expectations." "In no case where the system was brought under the influence of calomel, and ptyalism induced, did a fatal termination ensue."

"The greater proportion of cases not requiring bleeding, the calomel was in general at once had recourse to, in the dose of a scruple, sometimes with the addition of a grain of opium, to be repeated every four or six hours, according to the urgency of the symptoms, and continued till ptyalism was produced. This effect, if it was to take place, was commonly produced in twenty-four or thirty hours, when the calomel was intermitted, and a dose of castor oil was ordered.

The immediate effect of the calomel seemed to be to produce a flow of bile into the intestines, and the stools, which before consisted of blood, mucus, pus, and shreds, now presented the appearance of bile, and were spinach-looking.

As soon as the bile was observed in the stools, an immediate abatement of the symptoms took place—the vomiting and tenesmus were relieved—the pain of abdomen and heat at the pit of the stomach were diminished—and the patient was considered out of danger."

Notwithstanding the large quantities of calomel that were often given, severe salivation did not occur in more than two or three instances.

---

### Pleuro-pneumonia?

An interesting case is related by Dr. Hamilton, of Falkirk, in the last number of our Northern contemporary, which we deem worthy of notice. Thos. Hansie, aged 50, a strong-looking, hale man, who reported that he had enjoyed good health for 20 years past, complained of an acute pain in the lumbar and hypogastric regions of the left side, with tightness of the chest, strong quick pulse, some cough, tough sputa. Subcrepitating râle was heard in the lower part of the chest on that side—the respiration tolerably good in the rest of the chest. This was in the middle of July. By the fifth of August, the symptoms were so far subdued by the usual antiphlogistic means, that medical attendance was not deemed any longer necessary. A small blister was recommended to be kept on the side for a few days. Exactly a month after this (6th Sept.) Dr. H. was summoned to the patient, and found him in a very altered condition. He had become emaciated, with anxious countenance, and prominency of the left side. On examination, the ribs were less distinctly visible on this side, and he complained of pain here, and a distinct feeling of airy crepitation could be felt with the hand in the subcutaneous cellular tissue, between the second and third ribs, near the sternum. Here was a tumor, about the size of half an egg, cut in the long diameter. When this was gently pressed, it conveyed the feeling of air, contained immediately beneath the skin; and, when a greater degree of pressure was employed, there was a feeling, as if the air passed through a fluid into the chest. The tumor did not immediately re-appear when the pressure was removed. When the patient coughed, a strong impulse was sometimes conveyed to the hand through the tumor, similar to that felt, under similar circumstances, in hernia. A strong gurgling sound was heard through the stethoscope at this place, together with the metallic tinkling, during respiration. Over a large portion of the anterior part of this side, and except in the region of the heart, the same sounds were heard, but less strongly. The respiratory murmur was wanting in the whole of the left side, except faintly at the back part, near the spine. The sound, on percussion, was much duller in this side than natural. Catarrhal râles were heard slightly in the right side. The sputa were muco-purulent, and sometimes considerable in quantity. He was ordered to be cupped, and to take calomel and opium. *Sept.* 15*th.* Has been recruiting much during the last week—appetite good—perspiration less—can lie on the right side—expectoration diminished—tumor has disappeared. Has some diarrhœa still—the calomel and opium to be continued. 17*th Sept.* The sound on percussion is much clearer than it was. *Gargouillement* can be heard with the stethoscope about the middle of the anterior of the left side. The respiration is cavernous on a level with the fourth rib anteriorly. Beneath the clavicle, and close to the sternum, there is *gargouillement.* From this time he continued to improve, and about the middle of November he was able to work. On the 27th November, Dr. H. had an opportunity of examining the patient. In the situation formerly occupied by the tumor, the skin felt more dense than natural, so that the rib could not be distinctly traced. The respiratory murmur was distinctly heard on the posterior part of the left side; but the sound, on percussion, was still duller than on the right side. The man was then strong, and worked as a porter. On the 25th April, 1834, the man continued in good health.

Different opinions were entertained as to the precise nature of the lesion in this case. For our own parts, we have no hesitation in coinciding with Dr. Hamilton, that pleuro-pneumony was the first link in the chain—that sero-purulent effusion followed—that a communication took place with some of the bronchi—and that the matter very nearly made its way externally between the ribs. This, we believe, was the actual state of the case. The patient was fortunate to escape with his life.—Eds.

---

Cholera.—Dr. Hawthorne.*

The author of this pamphlet avers that "his mode of treatment differs from every other that has yet appeared be-

* A Dissertation, &c. Octavo, sewed, pp. 53. Oct. 1834.

fore the public:"—and again, that he has administered his remedies "with unparalleled, nay, with never-failing success." These being startling assertions applied to any disease, but particularly to cholera, we had curiosity to go through the brochure. We need not lay before the readers of this Journal any part of the history of the disease. Dr. H. thinks there are some reasons for believing that cholera is occasionally contagious; but still stronger ones for concluding that it is epidemic. In Belfast Dr. H. observed sporadic cases of intense malignity, long before the disease broke out generally —none of them appearing contagious.

"The horrible epidemic at length came over the town like a shower, filling streets and lanes, in almost every quarter, with wailings and lamentations. The attacks, on the night of the 12th, and on the morning of the 13th of June, were simultaneous, and widely scattered over the town, without any apparent communication."

The symptoms of cholera are detailed with sufficient accuracy—and to the RATIO SYMPTOMATUM we do not object. In respect to the pathology, we agree with our author, that death is caused by the loss of the serous part of the blood, (where the patient dies in the collapse, or anterior to reaction)—in other words, that he dies of serous hæmorrhage—a doctrine which we have long maintained, and to which Dr. H. can lay no claim. The methodus medendi of our author will be found in the following extract:—

"In offering a few remarks on the treatment of cholera, it may tend to facilitate my object to give a list of the *formulæ* which I have been in the habit of prescribing, and which have proved so successful. They are as follow:—

I. ANTISPASMODIC PILLS.

Camphor, half a drachm;
Opium, twelve grains;
Cayenne pepper, nine grains;
Spirits of wine, and conserve of roses, of each a sufficient quantity.—Mix.
To be made into a mass, and divided into twelve pills.

II. ANTISPASMODIC MIXTURE.

Sulphuric ether,
Aromatic spirit of ammonia,
Camphorated spirits,
Tincture of opium, of each a drachm;
Cinnamon, or peppermint water, two ounces.—Mix.

III. CORDIAL MIXTURE.

Brandy, or whiskey and cloves;
Ginger cordial, &c.

I shall first point out the steps to be pursued in the treatment of cholera, and then proceed to explain the *rationale* of the practice, and the *modus operandi* of the remedies.

I commence, therefore, with the malignant form of the disease.

The patient, on being seized with symptoms of cholera, should be immediately placed in bed in the horizontal posture. Should he be affected with watery purging, or serous purging and vomiting, a feeling of weakness, with a soft, feeble and variable pulse, and heat of skin below the natural temperature, he ought to get instantly six of the antispasmodic pills, and an ounce of the mixture already described. The medicine should be washed down with a dose of some cordial stimulant, such as a glass of ginger cordial, or of whiskey flavoured with cloves, or some such warm aromatic spice; or, if it should appear that the stomach would retain it, with a glass of whiskey or brandy punch, with essence of ginger in it. The punch to be made strong, and to be swallowed as hot as it can be let down. The body is then to be covered with additional blankets, and the usual means of communicating heat, such as jars or bottles of hot water, bags of roasted salt, or hot bricks, applied to the feet and different parts of the body, so as to restore the temperature, and to produce perspiration as quickly as possible. If one dose of the medicine shall be found insufficient to stop the discharges from the bowels, the half of the dose should be repeated, and, if necessary, an enema, composed of four ounces of boiled starch, with an aqueous solution of six grains of opium, or a drachm of laudanum, should be given, and its return resisted till it has had time to produce the desired effect. I have frequently seen the aqueous solution of the opium retained after the spirituous tincture had been rejected. *So much for whiskey injections.* If profuse perspiration be quickly produced, it will be seldom necessary to repeat the dose of the medicine. Should one dose of it fail, however, to accomplish the object, more, I again repeat it, ought to be instantly given. There is no alternative. The escape of the serous, or watery part of the blood, must be stopped, or it will assuredly destroy the life of the patient.

When the discharges from the bowels cease—when the pulse becomes full and bounding, and the body is covered with a copious warm sweat, in ninety-nine cases out of a hundred all danger is over, and the individual may be well next day. The hotter the skin becomes when covered with sweat, the better; and, I would add, the less the danger. The great secret in the treatment of cholera is to lose no time in stopping the discharges, and in exciting warm perspiration. This object should still be kept in view by the practitioner, no matter in what state he may find his patient. After giving such a dose of medicine as may stop the purging, his next effort should be, by the external application of heat, to produce a discharge from the surface. If, when called in, the heat be higher than natural, the perspiration will equalize the temperature; if lower, the heat will restore it; and if the body be covered with a cold, clammy sweat, it will change it to a warm one. It is remarkable how suddenly the pain of stomach, præcordial oppression, headache and spasms, if they exist, are relieved on the breaking out of a free perspiration; and what is of equal, if not of greater importance, is, that the vomiting in every case immediately ceases. I would here remark, that heat can be much more efficiently communicated by solid substances, such as I have mentioned, than by the hot air, or vapour apparatus. I have long since left that instrument aside as worse than useless. I would also observe that, in the application of external heat, a rational use should be made of the means, and I cannot see any necessity for increasing the temperature beyond what is merely required to keep up profuse perspiration.

As little drink as possible should be given till the perspiration has flowed freely for a few minutes, after which the stomach will retain it. The patient should then be indulged with copious draughts of sweet or rennet whey, warm toast-water flavoured with ginger, mint or balm tea, or of any such mild beverage. The necessity of particularly attending to this shall be afterwards explained.

The sweating, if the patient can bear it, should be kept up for twelve hours, and may with advantage be continued moderately even longer. Its duration, however, must be regulated according to the strength of the patient and the state of the pulse. After the first four or six hours, more heat need not be applied than what is agreeable to the feelings of the patient.

If the purging has been quick and violent, the bowels should not be disturbed for at least twenty-four or thirty-six hours after it has been stopped. It would be well in such cases, provided the patient be free from sickness at stomach, to allow the bowels to remain unmoved till the second day; they may then be opened by a mild laxative enema. Should, however, the state of the stomach, at the end of twenty-four hours, appear to render it necessary, it may be administered. In case, as it often happens, the patient on the second day complains of acidity in the stomach, and the bowels be confined, it will be proper to give him two table spoonsful of the following mixture every third hour, till a laxative effect is produced.

Calcined magnesia, two drachms;
Peppermint water, eight ounces;
Sweet spirit of nitre, an ounce;
Compound spirit of lavender,
Tincture of ginger, of each 2 drachms;
Tincture of colombo, half-an-ounce.
—Mix.

This mixture will neutralize the acidity in the stomach, and restore the healthy tone of that organ. It will also act gently on the bowels, cleansing the tongue and cooling the system, and will promote the restoration of the healthy secretion of urine, which is generally suppressed in that disease. It is, besides, a valuable remedy in the consecutive fever which so often succeeds a severe attack of cholera. If, however, in addition to acidity in the stomach, there be too great relaxation of bowels, the magnesia should be omitted, substituting aromatic spirit of ammonia, and camphorated julep, which, with the aromatics and bitters just prescribed, will remove the former without increasing the latter affection, and, if necessary, the tincture of opium may be added; or, if no pain, ten or twelve grains of the bi-carbonate of soda in a glass of water will answer the purpose.

The bilious vomiting, which so frequently occurs in the consecutive stages, may be relieved by a laxative enema. Bilious diarrhœa is to be treated with the cretaceous mixture, combined with suitable proportions of the tinctures of catechu and opium, and, if obstinate, by anodyne injections; the strength at the same time is to be supported with wine, chicken broth, and beef-tea. The healthy tone of the stomach should be restored by bitters and aromatics. As soon as the discharges from the bowels have resumed their natural appearance, the patient should be indulged with animal food—such as roasted beef, beef-steaks, or mutton-chops broiled. After the healthy functions of the body have been restored, the relaxation which has been produced by so much perspiration may be removed by sponging the body with vinegar, and rubbing with the flesh-brush or a coarse dry towel.

Great care should be taken not to allow the patient to get out of bed, or to stand in the erect posture, till the strength of the body and the healthy tone of the nervous system have been sufficiently restored. Fatal consequences have sometimes arisen from not attending to this precaution. In the Newtownlimavady Cholera Hospital, a woman, who had had a very favourable recovery from an attack of cholera, lost her life by imprudence in this respect. Contrary to the orders and remonstrances of the physician in attendance, she got out of bed, and while in the act of dressing herself in the erect posture, she suddenly fainted. The excretory vessels being unable to sustain the superincumbent weight of the fluids of the body, became dilated—the serum of the blood escaped into the bowels—the sphincter gave way, and she passed several quarts of fluid as clear as water. She was dead in two hours afterwards, having manifested all the symptoms of one who had been bled to death.

The milder forms of the disease are to be treated on exactly the same principle as the more malignant."

We believe our readers will be ready to remark that the principle of treatment laid down here is not so very different from any other hitherto recommended, as our author seems to think. There may not be any methodus medendi *precisely* similar in books; but the principle is familiar. The dose of opium prescribed by Dr. H. is much larger than that usually given. He remarks that, "the whole success of the treatment of cholera depends on the first dose. If the first dose be inefficient, the second may be too late."

We need not dwell on the consecutive fever. It must be treated on general principles. It is not of frequent occurrence, unless the patient has either gone into collapse, or approached very near to it. It is rarely met with unless there has been considerable purging—viz. serous hæmorrhage.

"My practice in cholera has not been in a corner. It has been considerable in six different counties in Ireland. In every part of the country where I have treated it, I have frankly explained to the medical gentlemen who co-operated with me, my views with regard both to the pathology and therapeutics of the disease, and every where, and at all times, the remedies I employed, and the doses of the medicines which I prescribed were open to the inspection of all."

The Appendix is chiefly occupied with criticisms on the modes of treatment adopted by others—and especially by his townsman, Dr. M'Cormack. These criticisms are conducted with great acrimony, and in exceeding bad taste. Several strong testimonials in favour of Dr. Hawthorne's mode of treatment are published in the Appendix. We shall insert one of them.

"*Board Room, Dungannon,*

*20th Jan.* 1834.

In consequence of recent discussions, in the public prints, on the subject of Cholera, the Board of Health feel called upon to state their decided approval of the mode of treatment of that disease, introduced here by Doctor Hawthorne; and, in confirmation thereof, give the annexed abstract of the number of cases treated, in and out of Hospital, on his plan.

Signed by order,

JOHN PEEBLES, Sec."

---

"FROTTEMENT OBSERVED IN PERITONITIS. Communicated by Professor BEATTY.

As any thing that can contribute to our means of discovering diseases of the heart, must be looked on as in the highest degree interesting to the practical physician, it has occurred to me, that a notice of some cases which have come under my observation, although not of disease of the heart, may serve to corroborate the views so ably set forward and maintained by Dr. W. Stokes, in his paper on the diagnosis of pericarditis, in the fourth volume of this Journal. It is there stated, that the opinion broached by Collin, in 1824, and which had gained no credence for nearly ten years, is founded in fact, and that we have a physical sign of inflammation of the serous lining of the pericardium, viz. a 'frottement,' or sensation of rubbing together of two uneven surfaces, distinguishable by the application of the hand, and by auscultation. The cases furnished by Dr. Stokes, in illustration of this point, are most interesting and instructive, and accompanied, as they are, by his judicious observations, must be considered as opening a new field in the departments of practical medicine and pathology. With a view to shew that similar effects are produced in the peritoneum, when that membrane is the subject of inflammation, I have been induced to forward the present communication.

In January, 1832, a woman aged 30, was admitted into my ward for the diseases of females in the City of Dublin Hospital, labouring under dropsy of the left ovarium. The tumor filled the abdomen from the pubis to the ensiform cartilage, and was remarkably hard and unyielding. A few days after admission she was attacked with severe pain in the belly and febrile symptoms, which continued for a week, and required the abstraction of blood, and other antiphlogistic treatment, before she was relieved; during which time a remarkable sensation was communicated to the hand when applied over the umbilicus and its neighbourhood. The sensation was that of a grating or rubbing together of two uneven and rather dry surfaces, and was rendered most evident by ordering the patient to take a full inspiration, thereby causing the abdominal parietes to move more freely over the surface of the tumor. By the application of the stethoscope, a loud and distinct 'frottement' was audible, extending over a space of about five inches in diameter, with the umbilicus for a centre. In a few days, the pain and inflammatory symptoms subsided, under the treatment employed, and with them, the sensation just described, and the audible phenomena altogether disappeared.

In the December following, I had an opportunity of observing similar effects, in the case of a young lady, who was under my care for excessive enlargement of the spleen. The tumor occupied the left half of the abdomen, dipping down into the pelvis on that side, and its anterior edge passed the median line of the body, particularly at the lower part, where it extended considerably into the right side. She was seized with inflammation of the tumor, and during its continuance, phenomena precisely similar to those described in the last case were perceived; there was the same creaking sensation when either the hand or the stethoscope was applied to the surface, and this entirely subsided when the inflammation and pain were arrested.

It would appear that this method of diagnosis of disease of serous membranes is applicable only in those situations, where one, at least, of the opposed surfaces is adherent to a solid resisting body. I am not aware that phenomena such as have been mentioned can be perceived in inflammation of the peritoneum, under ordinary circumstances, where the soft pliable walls of the abdomen are in contact with the mass of intestines; but when a large solid tumor comes to occupy the cavity, as in the instances above mentioned, the case resembles that of the pericardium with the heart within it, and similar physical signs of disease of the serous surfaces become apparent.

It has appeared to me that these cases may be employed as confirming the truth and accuracy of the diagnosis of pericarditis, and with that view I wish to record this brief notice of them."—*Dublin Journal, Sept.* 1834.

---

### Sir Charles Bell—the mighty Dead.

Homer and Virgil—Socrates and Cicero—the poets and the philosophers, of all ages and of every country, have eulogized the past, contemned the present, and bewailed the future! Yet the world has wagged on, and, in despite of these "laudatores temporis acti," many are so presumptuous as to think that improvements have taken place in most branches of science—nay, even in medicine. We have been led into some reflections on this subject by the perusal of some "lamentations," in the form of a clinical lecture, lately delivered by our talented and esteemed friend, Sir Charles Bell. He observes to his pupils, that *they* have not the advantages which *he* had—namely, of drawing information from a Black, a Monro, a Rutherford, a Gregory, a Cline, and an Abernethy. "We shall look around (says Sir Charles) in vain for such men."* The lecturer is led "to reflect upon the eminence of these men, and mentally to ask himself who are in their places now?" Sir Charles need not have gone far to find at least one who is worthy to succeed them. He has himself thrown more light on many obscure parts of an occult science, than any one, two, or three of the mighty dead which he has enumerated. The lecturer goes on to remark, that his pupils "have not the example before them, of men having the same *sway* over the profession" as those above-mentioned. Now we sincerely rejoice at this, instead of looking on it as a sign of the decay of genius, intellect, and industry, in modern times. The loss of this *sway*, which a very few exercised over the multitude of our profession, in days of yore, is owing to two causes—a more wide and a more *equal* diffusion of knowledge among the members of it. We say a more *equal* diffusion; for knowledge may be very widely diffused, but very unequally. Under existing circumstances, therefore, it is morally impossible for a *few* to outstrip the *many*, as in former times, when medical education was extremely imperfect in kind, as well as defective in quantity. The Blacks, the Monros, the Gregories, the Clines, and the Abernethys, shone in their times like stars of the first magnitude, or like moons among the twinkling luminaries of the night—

——————— velut inter ignes
Luna minores.

But we confidently aver that, were these mighty dead to experience a second incarnation, and run their course on earth again, they would not descend to posterity as THE Blacks, Gregorys, Abernethys, &c. of former days. No, verily. Nature is too economical to elaborate a dozen of Shakespeares Miltons, and Byrons at the same time. The medical luminaries already alluded to owed their celebrity to accidental circumstances, and the then state of medical society, rather than to any

* Med. Gaz. 18th Oct. 1834, p. 90.

towering intellect, rising high above the capacities of their descendants of the present day. But we will go much farther than this. We fearlessly assert, that the living surgeons of our own æra, including Sir Charles amongst the foremost, are infinitely *superior* to any and all of those which he has brought forward on the tapis. We do not say that they are superior by *nature*—but by the improvements of time. With the exception of a few rare examples of preternatural genius, exhibited once or twice in a century, we believe that Nature gives birth to an average of talent, in the great mass of society, *every year*. It is opportunity and industry that make the great differences in mental and experimental acquirements. Not that we think all men equal in point of natural capacity. On the contrary, there are hardly two men, in a large number, of equal calibre. But we maintain that the average ratio of talent is as great in the nineteenth as in the eighteenth, or in any preceding century; and that it is owing to the greater equilibrium of education now than formerly, that we have fewer prominent characters amongst us. We confidently predict that, as time rolls on, as education becomes still more diffused, and as the "stimulus of necessity" augments with the increase of population, this elevation of the few over the many will become less and less conspicuous. And it will be all the better for society at large. We shall have Coopers, and Brodies, and Keates, and Bells in our towns and villages—and many an expensive journey to the metropolis, for advice, will be saved in the twentieth century.

Sir Charles passes over LECTURERS in a parenthesis, and then makes a desperate lunge at *young authors*, of modern times. We may be supposed to have some knowledge of this subject; and we have no hesitation in asserting, that more trash issues from the pens of old than of young authors in the present century. Look at the far-famed prelections of the far-famed Abernethy —and ask yourself, Sir Charles, what is their use? If you speak the sentiments of your heart, you will say that they are as good as the "Arabian nights" for dispelling melancholy, and making us laugh in a dull Winter's evening—when we have taken no fees in the morning, and have been called to no patients during the day.

We are sorry to be obliged to differ, toto cœlo, from our estimable clinical lecturer, when he tells his pupils that they have not the example before them of men who have "as sedulously given themselves up to the improvement of the profession," as those of his day. We firmly believe, and as fearlessly maintain, that in no period of the Christian æra was there more strenuous, or more successful application to useful and practical science, in medicine and all its collateral branches, than in our own times. We do not indeed, addle our brains so much as our forefathers did, in the construction of theories; but we have far outstripped them in morbid anatomy—in diagnosis—in chemistry—in accurate clinical observation—in therapeutics—in operative surgery—in short, in every branch of practical and scientific medicine. In all these advances, Sir Charles Bell has stood in the foremost ranks—and his remarks, if serious, are little short of suicide, as respects himself, and fratricide, as regards his contemporaries.

---

OBSERVATIONS ON ERYSIPELAS. By EPHRAIM M'DOWEL, M.D. one of the Surgeons to the Richmond Hospital.

This forms the leading article of our Dublin contemporary for October last. It appears that the disease was epidemic in the Dublin hospitals in the beginning of 1834, and very frequently fatal. It attacked all ages and both sexes indiscriminately—the sick and the healthy—while almost every kind of injury was followed by the disease. It occurred after leeching and bleeding, venereal ulcers, and even the application of liniments to the surface. Surgical operations were so generally succeeded by erysipelas, that they were performed as seldom as possible. Like the epidemic influenza, it was characterized by extreme prostration of strength, rendering depletion precarious, and often dangerous, even where there was strong local inflammation, and the constitution was sound. In the progress of the epidemic, there was great variety in the numbers attacked at different periods. Sometimes it would be nearly extinct in the hospital, and then unexpectedly burst forth with renewed force. It was most troublesome in Easterly winds.

"The different cases of this disease met with by the writer, were referrible to one of three classes. In the first, occurring in healthy persons, and usually after injuries, there was much local and constitutional disturbance; the disease was often of the phlegmonoid form, but in other cases, the skin alone was acutely attacked. In the second class of cases, examples of superficial erysipelas, the redness was more diffused, frequently it was not very perceptible, and occasionally considerable patches of the skin were unaffected, passed over, as it were, by the disease; vesications occurred more frequently than in the more acute cases: this form attacked persons of weak constitution, or of unhealthy habits, in whom the biliary organs and stomach were deranged or diseased: the constitutional symptoms seldom ran high. Subcutaneous abscesses formed very insidiously, and frequently in considerable numbers, and, if not opened early, spread very quickly, death of the cellular membrane to a varied extent generally occurred, undermining the skin, and protracting much the cure. It was often necessary to examine a limb most carefully to detect these purulent depôts, the patient seldom being aware of their existence; the matter secreted was generally healthy pus. In one case of erysipelas of the head and face, succeeding ptyalism, slight pain, or rather uneasiness, had been complained of in the neck; there was no evidence of disease, but on examination after death, a very extensive *puriform* infiltration of the loose cellular tissue, about the trachea, thyroid body, and œsophagus, was found; it extended behind the pharynx nearly to the base of the cranium, and very probably was not merely an example of inflammation propagated from the skin, but also from the mucous membrane of the pharynx, which was also inflamed. In the diffused inflammation of the fauces in cynanche maligna, we have frequently similar formations of matter deep in the neck, from extension of disease from the mucous to the cellular tissue. In the third class of cases, the disease occurred in habits broken down by want, intemperance, age, or previous organic disease of long standing, and sinking occurred very early, unless prevented by stimulants. In such cases, there was less redness and heat of surface, the affection had more of the character of erythema, with a well-defined and raised irregular border; the pulse was quick and weak, the accompanying fever of the typhoid character, the tongue more or less loaded, dry and brown, or red, dry and scabrous near the apex, with elongated papillæ, the rest of its surface much loaded: this latter condition of the tongue generally indicated more or less of gastric irritation.

It is in this form of the disease, that on examination after death, we constantly find evidence of the previous existence of inflammation of the cerebral membranes, of the pulmonary, and of the intestinal mucous surfaces, the cutaneous affection being in fact but a small part of the disease. Severe rigors and diffuse inflammation of the throat, of a dusky red colour, with patchy deposition of lymph, and more or less of sloughing, as in cynanche maligna, preceded several severe cases of erysipelas of the head and face. When the scalp was attacked the inflammation generally became diffused, but occasionally, was limited pretty exactly to one half; when this got well, it was usual for the opposite side to become affected. Delitescence, or disappearing of the inflammation from one part to re-appear in another, was of frequent occurrence. There was a great tendency to the erratic form, particularly in the old, or in persons of broken-down constitution."

An attack of erysipelas was sometimes beneficial in chronic and obstinate cutaneous diseases, as lupus. When the epidemic was very prevalent, there was a remarkable tendency to diffuse inflammation of the digestive tube, without any accompanying erysipelas of the skin. The disease was then rapid—the prostration great. Dr. M'D. lost two patients in this way. The first patient was attacked with diarrhœa on the 4th of March, and died on the 12th, having exhibited tenderness on pressure of the abdomen, bloody stools, with mucus, &c. On examination, there was found diffuse erysipelatous redness of the mucous membrane of the stomach and bowels throughout —the redness being most intense near the termination of the ileon, where shreds of coagulable lymph were thrown out, abrasions and ulcers being also apparent.

Some practitioners, says our author, doubt or deny the existence of morbid appearances after death by erysipelas. He is not one of these. The fatal cases are generally those where the head and face have been affected, and where the last scene is characterized by coma. In these, he usually found much sanguineous congestion in the different tissues, from the skin to the brain—serous infiltration of the loose cellular substance under the scalp, and sometimes sero-purulent fluid—pericranium vascular and thickened—dura mater vascular—arachnoid sac containing a serous fluid—white or milky appearance of this membrane between the convolutions of the brain, with abundant sub-arachoid effusion into the cellular tissue of the pia mater—sinuses, cerebral veins, jugulars, and right side of the heart much congested—cerebral substance firmer or softer than natural, shewing numerous bloody dots.

In most cases of protracted erysipelas, inflammation of different portions of the gastro-pulmonary mucous membranes was found—especially of the stomach, bowels, and bronchiæ—the inflammation being most intense at the bifurcation of the trachea, with frothy, sanguineous effusion into the smaller ramifications. Gastritis was indicated by epigastric tenderness, vomiting, ardent thirst, loaded tongue, dry, red, and scabrous near the apex—rapid pulse, &c. the strength being very variable. Muco-enteritis was known by abdominal tenderness, of variable extent—tympanitic distention, diarrhœa, the pulse often but little disturbed, and the alvine discharges being mucous and gellatinous, with blood occasionally. When the disease spread to the large intestines, there were dysenteric symptoms.

Metastasis of erysipelas is of rare occurrence, but may take place. The author gives a case where erysipelas of the face suddenly disappeared, and was instantly succeeded by acute bronchitis, which quickly destroyed life. We have seen some instances of this kind; but more where the external erysipelas suddenly extends to the membranes of the brain, without entirely leaving its original seat in the skin of the face or head.

Our author could not see any proofs of contagion in the present epidemic erysipelas; but, from what he had seen in other years, he has no doubt that it is occasionally contagious, both in and out of hospitals. A letter from Dr. Brereton to the author corroborates this opinion, by his experience in the temporary fever and dysentery hospital. We think that few hospital surgeons or physicians can doubt the occasional contagiousness of erysipelas.

"The treatment adopted in the first class of cases, was general or local bleeding, frequently both were employed; incisions were made when there was much tension, heat, and throbbing: calomel, followed by some of the neutral salts, when the stomach was irritable, purgative enemata, and saline effervescing draughts. If the disease did not yield, and that symptoms of inflammation of the internal organs manifested themselves, calomel in doses of two or three grains, combined with small doses of opium, was exhibited every fourth or sixth hour, until the mouth was decidedly affected: and cases threatening a fatal termination, when once ptyalism was effected speedily ended satisfactorily. The mercurial affection of the mouth was in general easily controlled by a gargle of the solution of the chloride of lime or soda, with syrup and water; or by the free application of a lotion of nitrate of silver, twenty or thirty grains to the ounce. The vomiting (although in general sympathetic, in some cases obviously depended upon gastritis) was combated by leeching and blistering the epigastrium, and by the exhibition of calomel and opium, with abstinence from all food; drink in sips merely was allowed.

In traumautic erysipelas, emollient poultices and fomentations were employed; some were successfully treated with cloths dipped in cold water, and frequently renewed, or covered over with oiled silk to prevent evaporation: if the inflammation spread, nitrate of silver, in solution, or blisters were employed to arrest or extinguish it. In the second class of cases, general bleeding was inadmissible; leeches, blistering, or the nitrate of silver lotion were generally found sufficient to check the spreading inflammation. Internally, saline purgatives, with small doses of tartarized antimony, or, if the stomach was irritable, blue-pill, followed by saline aperients, and afterwards quinine in small doses, was employed; whenever lesion of the vital organs was threatened, mercury was steadily given to affect the system, at the same time supporting the strength by tonics and stimulants. In the third class of cases, it was necessary to prevent sinking by the early and liberal use of wine or porter, beef-tea, quinine, carbonate of ammonia, and opium; the latter was often necessary to check diarrhœa, but was contra-indicated when there was much cerebral or pulmonary congestion; it was then necessary to excite powerful and extensive counter-irritation, and to stimulate by sinapisms or blisters."

In respect to the local treatment of phlegmonoid erysipelas, every practical surgeon is now aware of the importance of early and free incisions, which speedily arrest the progress of the inflammation, give great relief to the patient, and prevent the sloughing of the fibrous and other tissues, as well as purulent infiltration of the cellular membrane. Puncturing is a bad substitute for incision, and the depth should be in proportion to the extent to which the inflammation has penetrated. In the great majority of cases, it will be unnecessary to go deeper than the cellular membrane. The bleeding is generally profuse, and should be watched, especially in people of intemperate habits or broken-down constitutions. "When (says our author) we observe the rapidity with which the blood flows, it appears pretty evident that there is no stagnation of blood in the inflamed capillaries." To us it appears just the reverse. The greater the engorgement and stagnation, the more rapid the efflux from the vessels when a channel is opened. "Qua data porta ruit"—like pent-up winds in the Eolian mountains. Where the disease has been neglected till sloughing occurs, lint, dipped in spirits of turpentine, or in gum elemi and turpentine, should be applied to the incisions, and the whole covered with an emollient poultice.

"Other local applications will also be found of much service in expediting the separation of sloughs and the processes of reparation, as equal parts of castor oil and balsam of capivi, the Peruvian balsam, or one part of the solution of the chloride of lime or of soda to six or eight parts of water."

When acute erysipelas is limited to the skin, our author recommends leeches. Dupuytren employs blisters. It is often necessary to exhibit tonics to support the strength, and rouse the depressed vital powers. Blisters have been employed with much benefit by our author.

"The local application of nitrate of silver, either in substance or in strong solution, so strongly recommended by Mr. Higginbotham, and by others, has been fully tried by the writer in very many cases. It should be applied not only to the inflamed parts, but also to the neighbouring integuments; it should produce vesication, or it is of little service; the smarting from the application lasts but a short time, and is soon followed by decided relief. On the next day, there is generally much diminution of the swelling, the blackened cuticle is much wrinkled, and desquamates in a few days; it aggravates the symptoms in phlegmonoid erysipelas, and also occasionally in the superficial form in very irritable habits. Some prefer this application to blisters, which, however, will often be found to succeed when the former has failed. Many object to these plans of treatment, but every practical man must be aware of the urgent necessity of arresting the progress of erysipelas, particularly when it affects the head and face, as an inflammatory state of the brain and its envelopes, accompanied by delirium, and ending in coma, may occur very early if the erysipelas becomes extended.

Lately, I have tried the plan of mercurial inunction, as recommended in the Lancet of July 14th, 1832, page 480, and of September, page 739, and upon the whole am led to consider that it is a most valuable application in this disease. To ascertain as much as was possible the value of this mode of treatment it has been employed nearly to the exclusion of other remedies, the bowels being merely regulated, and the diet attended to. In two cases where there was much sinking, tonics and stimulants were combined; in most of the cases, mercury applied in this manner affected the mouth. Understanding that this plan had been used in Mercer's Hospital, I applied to Mr. Reid for information, and was favoured with the following communication:—

'*York-street, Thursday, June 19th*, 1834.

I lose no time in giving you, from memory, the experience I have of the treatment of erysipelas by mercurial inunction in Mercer's Hospital. I have witnessed the practice both in the idiopathic form, and in the erysipelas consequent to wounds, &c.; it has been used in both species in the head, and also where the extremities have been the seat of the complaint. I think it is more than two years since this practice was introduced amongst us, and was suggested by the cases related in the periodicals, of its success in the hospitals of Paris. It certainly would appear to me, from a number of instances, to have considerable power in limiting the extent, and generally checking the progress of the disorder; two, three or four applications have usually sufficed, but the indications of medical treatment have been at the same time followed up. It has been remarked, that no case with us has died, on whom the practice was tried, and that if not in all, in the greatest number of instances, the patients were salivated: from this it would appear, that the absorbents in the diseased surfaces possessed an increased activity. We have now the case of an old man treated by inunction, admitted last Sunday; the erysipelas has disappeared, and the mercurial affection of the mouth is the principal cause of his present illness. This was a very unfavourable case; he received a contused scratch on the forehead a few days prior to admission; the face, scalp, ears, throat, and neck were much tumefied on his admission, and the right arm, from the wrist to above the elbow, similarly affected, though there was no local injury of the limb.'

Many cases are detailed, illustrating the varieties of erysipelas mentioned in this paper; but these we need not analyze. The whole paper is exceedingly creditable to Dr. M'Dowel—and to Dublin surgery in general.

---

### GASTRIC NEURALGIA.

A very distressing case of this kind lately came to our knowledge, which cannot fail to be interesting to many of our readers. A gentleman of high intellectual attainments, and one of the most successful authors of the day, had suffered most severely, for nine months, with an agonizing pain in the region of the stomach, which came on every day at breakfast time, whatever he ate, or whether he took food or not—and continued without intermission, but accompanied by the most distressing flatulence, till he had finished his dinner and taken a bottle of wine. It then ceased, and he was perfectly well, and in good spirits, the whole of the evening. He also slept soundly, except when kept awake by an anticipation of the pains which he was to undergo the next morning. The nature of the food made no difference; nor did an alteration of the hour of dining alter the period of the gastralgia. If he dined at his long-accustomed hour—six o'clock, and took a bottle of port-wine, his temporary security against the enemy was certain. An anticipation of the dinner-hour did not bring with it the relief expected. It may well be supposed, that a gentleman in his rank of life had had the best advice, and tried a variety of remedies. He resided in the country, and had never been our patient. He wrote a most pathetic letter, describing his sufferings, and imploring, for the sake of humanity, that we would suggest something for his relief. We prescribed the following :—

(No. 1—*Aperient.*)

℞. Decoct. aloes c. ℥iv.

Carb. sodæ, ʒj.

Carb. ammoniæ, ℈j.

Tinct. sennæ c.

Tinct. rhei comp. āā ℥ss.

Vini colchici, ʒij.

Misce fiat mistura, cujus capiat coch. ij. mag. primo mane, cum pauxillo aquæ tepidæ, et repr. dosis omni horâ donec alvus plene respondiat.

After the operation of the aperient, he was desired to take a table-spoonful of the following mixture every hour, till the pain ceased, or until the bottle was finished.

(No. 2—*Anodyne.*)

℞. Confect. aromat. ʒij.

Carb. ammon. ʒss.

Tinct. card. comp. ℥ss.

Liq. opii sed. ʒj.

Aquæ cinnamon. ℥iij. Misce. ut supra capienda.

We did not deem it prudent to alter his diet, as regimen seemed to have no influence on the complaint. We desired him, however, to change port-wine for brandy and water. We heard nothing from him for three weeks or more, when we received a letter, containing the following report.

" —— *Park, Nov.* 10*th*, 1834.
Dear Sir,—I ought long ago to have thanked you for your prompt and skilful attention to my distressing case; but waited till I could speak with certainty as to the success of your prescriptions. I have the pleasure to say that I am completely, or very nearly so, relieved from the flatulence, which was more difficult to bear than the pain. This relief I mainly attribute to your *warm aperient*, which I have taken every morning. I had got some temporary ease from the paroxysms of pain I described by a strong dose of morphium and strychnine, before your prescription arrived. It kept me dozing and sick for nearly 48 hours. I then took your aperient medicine, and it has been so effectual, that I am nearly myself again —certainly a very different creature from what I was three weeks ago. I have changed my port-wine for brandy and water, and enjoy my breakfast once more. I have not had occasion to take the anodyne marked No. 2 at all. I shall soon be in town to thank you in person for the great benefit I have derived from your able assistance."

The relief above described may or may not be permanent; but, even if temporary, it is of no small advantage. We desired him to strike out the colchicum, and persevere with the warm aperient. The gentleman is nearly 70 years of age, and of a very florid complexion, and, we believe, of gouty diathesis. We had never seen him but once, and that more than eighteen months previously to his application to us—when, in consequence of some official duties which we had to perform, this gentleman was somewhat incensed against us. This gastralgia was of a remarkable character, and we think it was occasioned partly by flatus and partly by some acrid secretion in the stomach itself. The regular periodicity of the attack, however, and the relief experienced by dinner and a bottle of wine, shew that the nerves were deeply implicated. Should we learn any more of this curious case, we shall state it to our readers.

### CREPITATIO MUSCULORUM.

In our last number, (October, 1834,) page, 452, we mentioned a curious case of loss of power in one of the lower extremities, with a remarkable and loud cracking of the muscles, when the limb was put in motion. We mentioned that we had recommended the patient, Lady W——, to make a tour on the Continent, more with the view of diverting her ladyship's mind, and improving her general health, than with much hope of restoration of power to the nearly paralyzed limb. She travelled through Holland—ascended the Rhine—visited the Brunnens, but did not bathe—made a tour through some parts of Germany, and also in Switzerland—returning by Belgium, and reaching London, after an excursion of eleven weeks. We preceded the patient in the same track, but having crossed the Alps into Italy, we did not happen to fall in with the party, or learn the result of the tour till recently. On calling at her Ladyship's residence, we were not a little surprized and gratified to find her walking about the drawing-room, without crutches—and almost free from lameness! We learnt that the power of the limb gradually returned as she pursued her journey, and indeed had been nearly restored before she reached the Brunnens—consequently she wisely travelled on, and recovered the use of her legs. We were now anxious to ascertain whether the crepitatio musculorum remained—and we found that it had nearly disappeared, but was now as loud as ever. Since we published the account, a medical friend called on us, and stated that he had the same crepitation in the muscles seated on the back of his neck. This we ascertained to be the case by applying the stethoscope. But the cracking is not near so loud in the case of the gentleman as in that of the lady. We have observed that this crepitatio musculorum is noticed by one of the Dublin lecturers (but his name has escaped us) as sometimes occurring in chronic rheumatism. The astonishing benefit, however, which has resulted from travelling exercise, and that in such an unpromising case as Lady W.'s, deserves record, as it may lead to other trials in similar circumstances.

---

### PLURALITY OF ASSISTANTS IN HOSPITALS.

This subject has lately given rise to some long and rather angry discussions at St. George's Hospital. Having heard that a strong opposition to the appointment of a second assistant-surgeon would be raised, we were not a little curious to learn the arguments on which such an opposition would be grounded. We therefore took the trouble to attend one of the Board-meetings, convened on that account, and must confess that we were far more surprised than edified by the ratiocination employed by the opponents of a measure which promised so much public utility. We expected that the arguments of the opposition would be based on some principle, and that no allusion would be made to any private or personal advantages or disadvantages accruing from the measure. Both lines of argument were adopted, however, on the occasion.—That which rested on principle (if principle it could be called) was futile, and very often unintelligible—that which based itself on personal reasons was somewhat injudiciously urged—especially when it proceeded from the individual personally interested. This part of the subject, however, we shall dismiss. In respect to the policy of enlarging the sphere of the surgical assistancy, by the appointment of two, instead of one assistant, we admit that the opponents of the measure laboured under the greatest disadvantages, inasmuch as no human ingenuity could devise anything like a rational objection to such a liberal and useful procedure. The only fault, indeed, which we find with the additional appointment is, that it is still too restricted. Instead of having a narrow crevice or loop-hole to such an institution, just large enough to admit a single individual, we would throw open a portly entrance, through which two assistants might enter, leaving an open stage and no favour for their professional exertions. Of all monopolies, that of a single assistancy to a public hospital is the worst. It shuts the door against merit—it prevents competition—and goes as far as it can to extinguish all energy and emulation in the monopolizing individual. ONE ASSISTANT SURGEON to such a noble pile as St. George's Hospital!! What would be thought of the man who convened a meeting of the inhabitants of Piccadilly, and, in a laboured speech, informed them that as there was one highly respectable surgeon-apothecary in the street, who was quite able and willing to physic all Piccadilly, long as it was, it would therefore be very beneficial to the said street and its inhabitants if they came to a resolution to have no other doctor there than the one in question? The proposer of such a measure would be considered crazy; and yet the adoption of his proposal would not be half so absurd, and not one quarter so injurious as the resolution, if adopted, of having but one assistant-surgeon to St. George's Hospital. The mal-contents of Piccadilly, when sick, might send to Clarges Street, Half-moon Street, or Curzon Street, for medical assistance, if they preferred a private to a parish doctor; but not so the inmates of the hospital. Whether the monopolizing assistants were idle, or ill, or ignorant—THEY had no choice! And when a vacancy for a higher grade occurred, we all know that the said assistant would, in ninety-nine cases out of the hundred, be the successful candidate.

One of the arguments against an additional assistant-surgeon, and which was adduced by the existing assistant, surprised us much—and, we have no doubt, astonished many of the auditors. It was maintained that such an appointment would injure him personally, and deprive him of half his reputation! Let us analyze this argument a little. If this personal injury should accrue, it could only be in one of two ways. He could only lose half his reputation by the superior ability *or* superior assiduity of his junior coadjutor. We venture to say, that the senior assistant of St. George's Hospital entertains no fear of being eclipsed, in respect of talent, by any assistant that may be elected to that institution. The other source of personal danger must lie in the superior industry of his junior collaborateur. Now, if the present assistant-surgeon permits himself to be outstripped by his junior in his labours to promote the utility and raise the character of the hospital, as well as of surgical science in general, we can only say that he deserves to lose—not half, but the whole of his reputation. Knowing, then, as we do, that nothing good, or great, or useful ever flowed from monopoly, that extinguisher of industry and moth of talent, we hail every competition in our public establishments as the forerunner and efficient cause of increased personal energy and augmented public good. In this paramount point of view, the new appointment at St. George's Hospital will be as beneficial to Mr. Walker as to his coadjutor or to the institution itself. He may be possessed of ample talent—but he shares the lot of mankind in ONE danger—that of idleness, if too secure in the prospect before him, and without the stimulus of emulation in his rear. We congratulate him, then, on the very event which he seems to deplore as a calamity.

We reiterate our opinion that every hospital, at all approaching the size of St. George's, should have at least two assistants to each department. It is idle to say that the work may be done by one. It will be far better done by two—besides the advantage of encouraging and training a greater number of young men for the service of the institution and of the community at large.

An instance, indeed, was related by Sir Benjamin Brodie, of an assistant-physician at St. George's Hospital being passed over, on account of incompetency—or supposed incompetency to fill the place of physician. But it is clear that the incompetency must be of the most glaring kind to induce governors to reject the only assistant in the hospital, and seek for a physician or surgeon out of doors. Such a stigma would be professional ruin to any assistant; but if there were two, the selection of a junior might be made with less injury to the one that was passed over for the time.

Again, we venture to draw the attention of the governors of the institution to the state of the out-patients. Scarcely a week passes that we are not mortified and chagrined at seeing patients rejected for want of room, and whose complaints require daily attention. Often are we induced to prescribe for and tend these poor people at their homes, when we can ill spare time from other, and certainly more lucrative avocations. Here would be an ample and fertile field of professional improvement; and we think that some plan by which the out-patient might be better provided with attendance, would greatly augment the number of subscribers to the hospital, since many are weekly disappointed by the rejection of those to whom they give tickets of admission. Pupils should never visit or have the charge of out-patients, except under the superintendence of some person of responsibility.

P. S. Since the above was written, we see that, by a great majority of governors, the principle of two assistant-surgeons has been recognized and approved, and an additional assistant accordingly appointed. We hope that an additional assistant-physician will soon follow.

---

ANATOMICO-PATHOLOGICAL RESEARCHES ON THE PNEUMO-GASTRIC NERVE. By J. T. H. ALBERS, of Bonn. WITH OBSERVATIONS ON DISEASES OF THAT NERVE. By Dr. HANKEL.

The state of integrity or disease of the pneumo-gastric nerve has, by reason of the important functions it presides over, attracted much of the attention of the more modern pathologists. In all the post-mortem examinations that came before him for nine years, Dr. Albers has never omitted to take notice of the state of this nerve. In the treatise before us, he mentions only those diseases in which the nerve had been most frequently involved.

In the autopsies of forty-seven persons who died of hooping-cough (some in the second, but most in the first stage of the disease), he examined both pneumo-gastric nerves in each subject, from their origin to the diaphragm. In forty-three there was no change in volume, colour, or consistence. Of the four others, who were scrofulous and lymphatic, the nerve of the left side was found in one slightly red, and that of the right side of the same colour in the three others. This redness was similar to that of the par vagum, in subjects victims to typhus fever, and was always on the side on which the patient commonly lay.

In seven cases of dothinenterite, the right pneumo-gastric was found red in two instances, the left once. This redness affected rather the tissue of the neurilema than the capillaries of the nerve. It disappeared by leaving the nerve some hours in cold water.

In the case of a robust man, æt. 27, who had been attacked in July with dyspnœa, anxiety, delirium, tetanus, and death, in the course of twelve hours, there was nothing presented itself at the dissection but redness and softening of the cervical portion of the pneumo-gastric nerve. It did not lose its colour by immersion in cold water, but by exceedingly slow degrees, and then was of a yellowish white. No doubt the colour and congestion may be produced on the dead body, by keeping the neck down and the head up, but in such a case the colour will be removed by cold water, and the nerve regain its original hue; but this did not occur in the case just quoted.

In fifteen subjects who died of tubercular phthisis, the par vagum was found developed in a remarkable manner; the right was much larger than the left, and in a more palpable degree than is found in the healthy state. This development of the nerve in phthisis is not rare; it has been observed by Swan and Descot.

In two cases of cancer of the œsophagus, the recurrent nerve was entirely destroyed by suppuration; in another case of perforation of the trachea and œsophagus, the vagus was partially destroyed, as was the recurrent.

In a case of medullary cancer of the mediastinum compressing the trachea and œsophagus, the nerve was developed so as to form a little tumor. The cancerous tumor surrounded the nerve. On cutting into its neurilema, there was found a tumor containing a substance similar to the cancerous one.

In a patient who died of intermittent croup, there were found tubercles in the left lung, tuberculous development of the cervical and bronchial ganglions, and intimate union of the pneumo-gastric of the right side with these ganglions. From these facts, Dr. Hankel attributes the attack of croup to the irritation produced by the softening of these glands, and the death of the patient to the paralysis of the pneumo-gastric nerve. He is also of opinion that many chronic affections of the chest are owing to organic alterations, tumors, &c. which compress and irritate the par vagum; and that if we find disease in the lung, we too often carelessly omit the minute examination of this nerve.

M. Andral relates a case, in the Nouvelle Bibliotheque, remarkable for its anatomical alterations. The patient was a young man of 24 years old, presenting the following symptoms: swollen countenance; œdema of the eyelids, and lower extremities; respiration short, confined, and depending so much on the pectoral muscles, that the lungs seemed paralyzed; inability to remain in the horizontal position: lips and alæ nasi blue; the stethoscope indicated no disease of the heart. Death succeeded suddenly to a fit of dyspnœa. On examination after death, a portion of the par vagum was found enveloped in tuberculous glands, below which the substance of the nerve was in a state of cartilaginous induration.*

* Dublin Journal, Nov. 1834.

### Remarks on Tubercular Phthisis with Symptoms of Obstructed Circulation. By R. Poole, Esq. Assistant-Surgeon, 32d Regt.

[Dublin Journal, Nov. 1834.]

The perusal of this paper brought to our minds many mortifying recollections of false prognoses delivered, even in a very late period of our professional career, respecting affections of the chest. Frequently have we come to the conclusion that our patients laboured under cardiac disease, when dissection proved that it was disease of the lungs. We have often thought that the "confessions" of a medical practitioner, who had arrived at mature age and extensive practice, would form one of the most interesting volumes ever offered to the public. By "confessions," we mean a candid acknowledgement of errors committed, and false opinions formed. We despair, however, of finding a Rousseau in Physic. Few have courage to acknowledge all the mistakes which they have made, either in diagnosis or practice, and thus thousands of rocks and shoals are left unmarked by flag or buoy, to wreck the unsuspecting voyager.

Two cases are detailed by Mr. Poole, in which the usual phenomena of tubercular phthisis were either obscure, or masked by symptoms of cardiac disease. In the first case, the predominant features were, dyspnœa, palpitation, puffy countenance, some cough, muco-purulent expectoration, bulimial appetite. On examination after death, the heart was found enlarged, and the whole of the *left* lung, as well as the two upper lobes of the right lung, were found completely infiltrated with tubercular matter. There were one or two excavations in the summits of both. There was passive dilatation of the right ventricle of the heart—the left chamber slightly hypertrophied.

The second case is far more minutely detailed, as it was constantly under Mr. Poole's care. The patient came into hospital, in November, 1833, with symptoms of inflammatory fever. He was remarkable for the purplish colour of the cheeks and lips. He went through a course of mild fever, characterized by a remarkable tendency to asphyxia, as well as lethargy. He was convalescent by the middle of December, and allowed to leave the hospital. On the 22d January, 1834, he was again admitted. His face was much flushed and very turgid—great anxiety —full hard pulse—hurried respiration —dry cough—no decubitus difficilis—acute pain over the whole chest on pressure. There was dulness on percussion, in the left submammary region, the rest of the chest sounding clear—respiratory murmur clear and distinct throughout both lungs—considerable impulsion of the heart, attended with some *bruit de soufflet*. Nearly 50 ozs. of blood were taken from the arm, and three quarters of a grain of acetate of morphine administered. He slept profoundly that night, and in a few days he was so far recovered as to leave the hospital again. On the 7th February he was readmitted, with pains of trunk and extremities, and cough. He recovered and was discharged on the 15th. He did duty till the 12th March, when he was once more admitted, suffering from slight pains of shoulders and chest —cough—expectoration. On the 17th he presented the livid complexion—circulation slightly accelerated—pain in the left upper breast and shoulder—considerable cough—little or no loss of flesh. On the 19th he complained of much pain in the epigastrium and abdomen generally, which is slightly tumid—left cheek very livid—pulse 100 —cough and expectoration diminished. The reports are carried on from day to day, with great minuteness, so that we cannot follow them. The symptoms were often anomalous and puzzling, we admit; but we confess that we should have entertained little or no doubt that a disorganizing process was going forward in the lungs. We shall just quote one day's report, as an example.

"26th March. Passed another bad night, muttering to himself, and otherwise irrational; vomited some last night and this morning after drinking some barley-water; says he is better this morning; his appetite is less voracious; breathing much more rapid; pulse very small and soft, still upwards of 100; a good deal of cough; expectoration still copious; the right hand has become livid; decubitus chiefly on the left side, but he can lie on his back; is unable even to turn on the right without producing cough, and increased dyspnœa; heat moderate; right side less defective in resonance, but the respiratory murmur here is still obscure; along the spinal edge of the scapula, on this side, a fine mucous or subcrepitating rale attends the respiratory murmur; left side affords a dull sound on percussion only at the cardiac regions; the whole being at this side, except a small portion at its summit, affords also a distinct subcrepitating rale; impulse of heart very inconsiderable; sounds much diffused."

He died on the 6th of April, and the chest presented the following lesions.

"The lungs did not collapse on the chest being opened. The anterior fringes of the right, and the anterior portion of the upper lobe of the left, were the only parts that presented the natural colour of the lung; all the rest was of a deep livid hue. The parts above-mentioned were alone inflated when air was thrown into the lungs. The right lung was in every portion of it closely infiltrated with small, round, crude tubercles, not one of which presented a trace of softening. The intervening space of the parenchyma, with the exception of the anterior fringes, was gorged with dark-coloured blood. There was not the least appearance of hepatization, but the blood did not escape on cut portions of the lung being pressed. No part of it sank in water. Its texture was not granular, and it readily broke down under the finger. The fringes were equally well supplied with tubercles; some of the bronchial glands were infiltrated with a soft cheesy matter. The left lung was also closely studded with the same kind of tubercle observed in the right; the lower lobe and the posterior surface of the upper were gorged with blood. In neither lung was there any aggregation of the tubercles, all were distinct from each other; the heart appeared large

*in situ;* its envelope contained eight ounces of a lemon-coloured fluid. Both ventricles were gorged with coagulated blood: the right was greatly dilated, and its parietes very thin; the walls of the left were not thick."

Mr. P. observes that the cases narrated "present few features in common with the ordinary examples of phthisis." On the contary, he says, "their whole course was attended by phenomena the very opposite to those the disease in general exhibits." The peculiarities of these cases, we think, with the author, may be fairly attributable to the great infiltration of tubercular matter into the lungs—the necessary obstruction to the circulation there—and the circumstance of the tubercular matter not making its way out in the form of purulent expectoration. The heart, in both cases was diseased; but not to that extent as to be the cause of death.

### Phlegmasia Cœrulia Dolens.

A middle aged woman was attacked, about three weeks after delivery, with symptoms of an intense peritonitis, for the removal of which the most active measures were necessary. These were succeeded by a state of extreme prostration, and after a few days, intense and universal bronchitis set in; so severe as for a considerable time to leave scarcely a hope for the recovery of the patient. It became necessary after the first few days of this attack, to have recourse to the free use of stimulants, consisting of wine, the decoction of senega with carbonate of ammonia, and the employment of blisters to the chest. The chest affection gradually subsided, and the patient appeared convalescent, when she was suddenly attacked during the night with violent pain in the left leg and thigh: and on the next morning the affected extremity presented all the appearance of acute general phlegmasia dolens, with the exception of the colour The limb from the groin downwards, was universally and equally enlarged. It was hot, elastic, exquisitely sensible, and deprived of motion. There was little or no swelling of the glands of the groin, nor was there any apparently cordy state of the saphena; but the remarkable circumstance was the colour of the limb, which was a deep purple hue, in some places almost black, and presenting more or less of a mottled appearance. This coloration was universal, and presented a most extraordinary contrast with the rest of the body.

The patient was treated by leeching, and the free exhibition of calomel and opium, the strength being supported by nutritious broths. The discoloration of the limb disappeared in the course of a few days, and her recovery was rapid and complete.

It was remarked in this case, that during the first days of the inflammation, no pulsation could be felt in the femoral artery at the groin. But this returned with the subsidence of the swelling.*

### On Transverse Malposition of the Head, as a Cause of Difficult Labour. By W. F. Montgomery, M. D. Professor of Midwifery to the King and Queen's College of Physicians, Ireland, &c. &c.

This paper forms one of a series in our highly respected contemporary of the Sister Isle. In a former number of the same Journal, Dr. M. insisted strongly on the necessity of an intimate acquaintance with the mechanism of labour, and especially with the exact relations which the different parts of the child's head obseive with those of the pelvis, during the process of natural parturition—a knowledge which often prevents the necessity of having recourse to instruments. Dr. M.'s observations, and the two cases in illustration, are so important in themselves, and so incapable of abbreviation, as to language, that we think a couple or three pages of this Journal may be well dedicated to their insertion.

* Dublin Journal, November, 1834.

"I presume I may take for granted, that every one engaged in midwifery practice, has from time to time met with cases in which, while every thing seemed favorably circumstanced, and the labour was apparently proceeding expeditiously to its termination, the head has become suddenly stationary in the *cavity of the pelvis,* and there remained for many hours, or perhaps until a necessity has arisen for adopting instant means of delivery; and this too when there really existed no deficiency of space to prevent its free passage. The occasional cause of this species of arrest, I believe to be not at all generally understood, which I shall now endeavour to elucidate, by a description of a particular kind of displacement to which I have been in the habit in my lectures, of applying the name of *transverse malposition;* and by the detail of one or two cases in which its detection afforded an opportunity of giving instant and complete relief, and happily terminating a severe and protracted state of suffering.

I may just premise, that in the most perfectly natural labour, the head enters the pelvis with its longer axis in coincidence with one of the oblique diameters of that cavity, and with the chin pressed up close upon the chest, until the vertex has descended so low as to press upon the soft parts forming the floor of the pelvis; the occiput then begins to advance towards the arch of the pubis, and the face retreats towards the hollow of the sacrum; next the chin recedes from the chest, and the occiput issuing from under the pubis, the head escapes by revolving as it were on a pivot under the anterior wall of the pelvis, so that in this way the head passes through the bony and unyielding chamber of the pelvis, in such a position, that it occupies the least possible dimensions, and the departure of the chin from the chest, which immediately requires great accommodation, does not take place until the occiput having cleared the confines of the pelvis has *unlimited* space to allow of its escape. But occasionally this felicitous arrangement is disturbed, and complete arrest of the head produced by the deviation which occurs thus; the head having entered the cavity of the pelvis in the position already described, the occiput, instead of moving forwards towards the pubis, recedes towards the spinous process of the ischium, and the face, instead of retreating towards the sacrum, falls into the space between the opposite spinous process of the ischium and its tuberosity; and the chin having receded from the chest, the head is placed with respect to the outlet in the most unfavourable manner possible, since it presents to it the greatest possible dimensions which it is capable of assuming, its longest diameter resting its extremities on the opposite tuberosities of the ischia, while at the same time the parietal bone rests on the lower part of the sacrum and coccyx, so that the head is in the condition of a ball supported on three nearly equi-distant, solid, and immoveable points; under which circumstances the action of the uterus, however vigorous, seems totally incapable of either changing the relations thus established, or of effecting the delivery, while they continue as they are. On examining, the finger will pass readily between the head and the pubis, and also posteriorly except at the point of the sacrum, but there, and opposite the tuberosities of the ischia, the head it felt to be closely locked; the anterior fontanelle is found to be in the centre of the presentation, and the sagittal suture can be traced exactly across the outlet from side to side. How completely and how long this malposition will resist the most powerful action* of the uterus, and how easily may it be rectified, will appear sufficiently from the subjoined cases; from which also it will be seen, that this difficulty (occurring as it does at a time when from the advanced position of the head, and the state of the perineum and other soft parts, delivery is momentarily expected) is likely to be productive of extreme embarrassment to the attendant, the more especially as the previous birth of full grown chil-

* "Valentissimi dolores nihil proficiunt."—RŒDERER.

dren, or even the circumstances of the case, may of themselves afford proof, that there is no natural deficiency of space, since the head may without difficulty be raised from its situation, to which however it immediately returns, and will there remain until the necessary rectification is effected, which should be done in the following manner. Apply two fingers along the junction of the parietal and frontal bones anteriorly, then *in the absence of pain* press up the forehead and push it backwards towards the sacrum, and there retain it till the access of the next pain which will in general complete the rectification, and the delivery is speedily accomplished, at least it so happened in the instances which came under my observation. It is scarcely necessary to add, that if, during the descent of the head, a tendency to this malposition be observed, we should at once endeavour to prevent its occurrence, by adopting the means already pointed out as suited for its correction when established; and this I have succeeded in effecting in a few instances.

Case 1.—On Wednesday, July 15, 1829, I was requested to see a patient with the late Mr. Gregory, and Dr. Carter; labour had commenced on the previous Monday evening, and proceeded actively on Tuesday; by six o'clock, P. M., the perineum was distended, and the head apparently on the point of being born: in this situation, however, it remained at nine o'clock, A. M. of Wednesday, when I saw her; although the uterus had continued to act most energetically the whole of the intervening time, and the soft parts were perfectly relaxed. It was the patient's second labour; she was young, healthy, and well-formed, and had about eighteen months before borne her first child, which was full sized, after an easy labour of about five or six hours, so that deficiency of space was not probable. On examination I found the head pressing on the perineum, I could pass my finger quite easily between it and the symphisis pubis, but at the sides there was no room at all: the anterior fontanelle was in the centre of the passage, the sagittal suture coinciding with the transverse diameter of the outlet, and the occiput turned to the left ischium: the uterus was acting violently, but produced no other effect upon the head than that of pressing it a little downwards during each pain, on the cessation of which it immediately resumed its original situation. Under these circumstances, I proposed manual rectification of the displacement evidently existing; and having applied my fingers, as already described, along the side of the forehead, I raised and pushed it backwards, towards the right sacro-iliac symphisis, in the interval between two contractions of the uterus; I there retained it, and on the accession of the next pain, I repeated the pressure backwards, when the forehead immediately glided to its proper place, at the same time the vertex moved forwards to the arch of the pubis, and *in about two minutes* the delivery was completed by the birth of a fine healthy child.

Case 2.—On the 14th of January, 1834, while lecturing at Sir Patrick Dun's Hospital, I received an urgent message from Mr. Dunlop, requesting my assistance in a case of obstructed labour, which he had been called to see, and in which, from the extreme violence of the uterine action, he apprehended rupture of the uterus, if the head was not speedily extricated; it was the woman's fifth labour, the four former having been short, and in every respect favourable. In the present instance, symptoms of labour had come on the evening before, the pains had continued gentle through the night, but towards morning they became more active, and at half-past seven o'clock the head was pressing upon the perineum to such a degree, that its exit was momentarily expected, but there it remained, without any further advance, when I arrived at half-past eleven, although the uterus had been, during the whole of the intervening four hours, acting incessantly and so powerfully that its rupture was with great reason apprehended. On examination, I found the perineum and soft parts protruded by the head, they were unusually relaxed and yielding; the head lay across the outlet of the pelvis, with the occiput resting against the tuberosity of the *right* ischium, and the forehead against the left, having probably descended in the second position; there was abundance of space between it and the pubis, *and it could be easily raised into the cavity of the pelvis*, but the next pain instantly forced it back to its resting place, and when there, the uterine action, although so strong, had no further effect on it whatever. I immediately adopted the same mode of rectification as in the former case, by elevating the forehead, and pushing it round towards the sacrum, when it almost instantly assumed its proper relations under the influence of a pain, *and the very next contraction of the uterus expelled it;* the body immediately followed, and the delivery was completed in *less than two minutes* from the time of effecting the change of position in the head. The child was alive and vigorous. I was fortunate enough in this case to have the valuable assistance of my friend Dr. Darley, who happened to be with me when I was sent for.

With reference to these cases, it is to be observed, that the subjects of them were women who had already borne children without any difficulty, and that there was evidence at the time, from the circumstances of their cases, that there was abundance of space, as the event fully proved; and yet the obstacle created merely by this kind of malposition of the head was such as to resist, in the one instance for *fifteen hours*, and in the other for *four*, action of the uterus, so powerful that it effected the delivery almost the very instant that the displacement was corrected.

As to the cause of this malposition, I am not prepared to offer any satisfactory explanation, nor is it, as far as I can see, a matter of the least consequence. The idea of Levret, that such misplacements of the head were caused by the situation of the placenta, is so unsupported by either facts or reasoning, and is indeed so fantastic, that I think we may dismiss it at once without further consideration. Neither is the hypothesis, which would explain them by obliquity either of situation or action of the uterus, in any degree more satisfactory. Rœderer ascribes some such deviations to misdirection of the shoulders, which he supposes in such cases to be placed across the smaller diameter of the brim; that this may be so occasionally is not improbable, but in the particular species under consideration, I think it certainly is not the case, because we can so completely correct it, merely by changing, and slightly too, the position of the head without moving the child's body at all. One thing, however, is certain, that when the malposition has taken place, the chin of the child has receded from the chest, and the forehead has sunk as low as the occiput, and that its re-elevation is essential to the rectification, and must be accomplished before delivery will take place; for while the transverse position continues, the natural efforts will not be sufficient, the forceps will not answer our purpose, and turning is out of the question, so that if the real nature of the case be not recognized, recourse will be almost certainly had to the appalling operation of cephalotomy, and a human life unnecessarily sacrificed. It seems very reasonable to suppose, that an unusual projection or curvature of the spinous process of the ischium might have the effect of producing this accident, because, under such circumstances, the forehead being prevented from gliding backwards, and being still acted on by the uterine contractions, would almost of necessity, be forced downwards into the situation where we find it in such cases, and the occiput would, of course, assume the corresponding situation at the opposite side of the outlet."

Our obstetrical brethren, now constituting nineteen-twentieths of the profession, will feel interested in the perusal of the foregoing observations.

---

### Incipient Phthisical Symptoms relieved.

Dr. Forsayth, of Templemore, in the county of Tipperary, has forwarded a case of what appears to have been commencing phthisis, in which the symptoms were perfectly relieved. The issue is of course uncertain, and melancholy experience forbids both Dr. Forsayth and ourselves to indulge in confident hopes of the result.

The patient was a young gentleman, aged 18, whose sister had died of phthisis previously. We need not enumerate his symptoms, which were highly characteristic, and such as would readily occur to the mind of the intelligent practitioner. The treatment was as follows.

"I confined him to *strict vegetable* diet, except milk—no vinous liquor whatsoever: tartar-emetic ointment to the affected side of the chest—bowels kept free by aloes and hard soap—and tinct. digitalis, 10 gtt. ter die, increasing 1 gtt. each dose, until the pulse fell to 56, at which I kept it.

The digitalis agreed, and he began slowly to amend. Pulse 56—respiration 24 to 26—cough declined—dyspnœa disappeared—no night-sweats, and less pain in the chest; and in two months nearly all the symptoms left him. Strength and appetite returned—tartar-emetic ointment, applied at three months, took away some remaining pain—animal food allowed—digitalis discontinued, and is now without any complaint, except slight uneasiness of breathing if he runs any distance."

After this, Dr. F. allowed his young patient some animal food. The method pursued by our correspondent was judicious. If, formerly, physicians kept phthisical patients too low, we fear they now fly to the opposite extreme, and aggravate inflammatory action by tonics and by food. They should recollect that, in some instances, tubercles originate in inflammation—that in all, their development is accompanied, in certain stages, by inflammatory action. Even when the pulse is most quiescent, and the skin most cool, a slight change of weather, or increase of food, will suddenly be followed by an access of fever, pain, and inflammation. Few cases require more judgment on the part of the medical attendant than phthisis; unable to cure, he may aggravate or relieve.

Dr. Forsayth sends us an interesting notice of an ossified tunica vaginalis testis.

"I have also in my possession, found in an old man, who died of fever here, a dried preparation of an ossified tunica vaginalis testis. Surface uneven—white fracture earthy—testis, tunica vaginalis testis, and albuginea healthy—two or three spots unossified, and transparent like glass. I believe there are two in London—none in Dublin Surgical Museum. It weighs ℥j. ʒiij. apothec. contained water:—Length, 3½ in.—breadth, 2 in.—greatest thickness, ¼ in. Soluble in nitric and muriatic scids with effervescence, leaving an equal size of gelatine or cartilage. By the blow-pipe it blackens, and remains white, and is entirely soluble in the acids."

---

### Case of Ununited Fracture, cured by Friction and Pressure.

In a late Number of this Journal, we noticed some remarks of Sir Benjamin Brodie's on the treatment of ununited fracture. Those remarks were founded on a case of ununited fracture of the tibia, for which Sir B. Brodie employed the seton. The present fact may be added to the collection at which that eminent surgeon glanced. It is related in the Number of the American Journal of the Medical Sciences, for August of this year, by the gentleman under whose notice it occurred—Dr. Parrish, jun. of Philadelphia.

*Case.* An athletic man, aged 27, fractured the left humerus in March, 1833. He immediately applied to a surgeon in a neighbouring town, who carefully adjusted the fragments, and placed the limb in splints; the injured parts being but slightly painful, the first dressings were allowed to remain undisturbed for about three weeks, when other splints were substituted, and continued on the limb, with occasional alterations, for three months. At the end of this period, finding no improvement, his physician advised him to seek further advice.

On removing the splints, the limb was found to be much reduced in size, its muscular power was obliterated, and its capillary circulation feeble. He was advised to lay aside the splints and bandages, to use the limb moderately, and to keep up a steady system of external frictions. This plan was pursued till the Autumn, when no amendment was perceptible, and he placed himself under the care of Dr. Parrish and Dr. William Ashmead. The following was then his state.

"On a careful examination of the parts, we found an unusual obliquity in the fractured portions, the surfaces exposed being not less than three inches in extent, the edges of these surfaces, the rounded extremities of the fragments, and the crevice separating the opposing surfaces of the fracture, could be distinctly traced by the fingers. This examination was rendered peculiarly satisfactory, in consequence of the emaciation and flaccidity of the limb.

Owing to the remarkable extent of the fracture, and the loss of muscular power in the arm, the fragments, which in a more vigorous state of the surrounding parts, might have been kept in apposition, were separated from each other to a greater or less extent, as they were influenced by the position of the limb. When the fore-arm was flexed upon the arm, in the usual attitude for fractured humerus, the surfaces of the fragments were separated throughout their whole extent, but more particularly at their upper portion—and it was only in one position that their apposition was effected.

The limb being placed in that position, which we found upon trial effected a perfect coaptation of the parts, the upper and lower portions of the broken bone were grasped by the hands, and a firm, gliding motion communicated, so that the surfaces could be felt rubbing upon each other. This process was continued for several minutes, and the limb was then secured in this position by light dressings in an angular box—a piece of thin board being firmly bound over the seat of fracture.

This process was repeated for several successsive mornings, and was performed by Dr. Ashmead or myself: the few first trials excited but little sensation in the fractured surfaces, though the force used was as great as we could command. In a few days, however, the patient began to feel pain, which increased at every repetition of the process, until it became acute. The fractured ends were less moveable, heat and action were re-established in the limb, and we were obliged to diminish the frequency and severity of the friction.

In about a month, bony union became evident at the lower extremity of the fracture, which proceeded rapidly, and so agglutinated the lower portion as to prevent the necessity of the box: shooting pains were frequently experienced in the limb, and any attempt to disturb it produced considerable suffering. Under these circumstances, we declined interfering with the salutary operations of Nature, which proceeded most happily. In about two months after the commencement of the practice, we had the satisfaction of observing, that a firm bed of callus was thrown out over the whole surface of this extensive fracture.

The muscles soon acquired their accustomed volume and force, and the man has since been pursuing his laborious occupation."

The employment of friction is not new; indeed in most cases, where union after fracture is sluggish, this, combined with pressure, is the first expedient usually had recourse to. The case of Dr. Parrish is deserving of notice, from its shewing how much may sometimes be effected, by a steady perseverance in a simple plan of treatment.

---

### Hypertrophy of the Mammæ.

We have lately, upon several occasions, adverted to this rather curious affection. The following is another instance of it. It is related in the same Journal from which we have extracted the preceding case, by Dr. Huston, resident physician in the Philadelphia Alms-House.

*Case.* A coloured girl had, from early life, been an inmate of the Philadelphia Alms-House. At the period of puberty, in addition to the ordinary changes that occur, her left breast was observed to enlarge disproportionately, and, in four months after she attained her fourteenth year, it had reached a very formidable size. The complaint, or rather the unnatural growth, continued to increase until about the age of fifteen years and a half, when both breasts enlarged with fresh and great rapidity. A laced jacket was required to support the ponderous mass, yet the girl's activity was apparently unimpaired. She appears to have menstruated only once, and then to a slight extent. On the 14th of April of the present year, she was received into the Alms-House, having been in service for some time previously. She had just completed her sixteenth year. Sloughing had attacked the integuments, on the inferior surface of the left breast, in consequence of a contusion recently received there. There was much pain and febrile irritation. On the 18th, the whole surface of both mammæ evinced a disposition to sphacelate. Low fever had set in, and it was evident that the girl would die; she did die, upon the 22d.

*Dissection.* "Externally the mammæ presented the appearance of two large oviform masses, rising above the clavicle, and extending below the umbilicus. No traces of the nipple could be detected, having been completely embedded by the enormous distention of the parietes of the mammæ. On measuring each breast, the dimensions were ascertained to be as follows :—

*Right Breast.*

| | |
|---|---|
| Greatest circumference . | 34 inches |
| Lesser do. ...... | 18 do. |
| Weight .............. | 12 lbs. |

*Left Breast.*

| | |
|---|---|
| Greatest circumference.. | 42 inches |
| Lesser do. ...... | 26 do. |
| Weight .............. | 20 lbs. |

On removing the right breast and exposing its interior, instead of a mass of disease or an accumulation of fluid (as a superficial examination might have induced the belief), it proved to be a mere hypertrophy of the organ unconnected with structural disease. The adipose and cellular tissues, as well as the whole glandular apparatus, were enormously enlarged, but no appearance of disease or exudation of fluid were perceptible. In short, a healthy structure was found, whose only anomaly was its mammoth proportions.

On examining the organs of generation, the ovaria were found to be larger than natural, and apparently diseased. The uterus did not exceed the ordinary size of females at her age, but two-thirds of its inner surface was coated with a dense covering of coagulable lymph. The muscular system was moderately developed."

Dr. Huston remarks, that the enormous augmentation of the mammæ does not seem to have diverted, in a material degree, the stream of nutrition from the other organs of the body. This may be, and apparently is correct; yet one reflection may induce us to suppose, that considerable debility of the system was produced. In the present instance, a contusion of the skin of the left mamma gave rise to sphacelus, more extensive and more fatal than a similar injury in a healthy person, on a healthy part, would probably occasion. A nearly similar case occurred at St. George's Hospital. A girl travelled up to that institution from the country, on account of enormous hypertrophy of the mammæ. On her journey, she slightly chafed the integument of one with the bone, we believe, of her stays. In a day or two after her admission, this abrasion gave rise to erysipelas, which ran into sloughing with such furious rapidity, that in four and twenty hours she was dead.

Morbid growths and morbid alterations of structure, are short-lived; that is, they evince a disposition to ulcerate and slough. They have not the vitality of the healthy tissues, but inflame more frequently, die more readily, and have less capability of resisting natural changes and external injury. It is probably owing to an analogous cause, that the erysipelas and sphacelation, in these two examples of hypertrophy of the mammæ, proceeded to the fatal extent which has been described.

---

Substance of a Clinical Lecture on Two Cases of Hydrophobia, delivered at the Charing-Cross Hospital, &c. By J. T. Pettigrew, F.R.S. &c. Octavo, pp. 35. London, 1834.

By a singular, yet not very unusual coincidence, two cases of this comparatively rare disease were witnessed, in quick succession, in the Charing-Cross Hospital. They received the close attention, and exercised the ingenuity, of Mr. Pettigrew, and they formed the subject of some discussions at the Westminster Medical Society, and the occasion of the present pamphlet.

The cases themselves were most assiduously watched, and are most faithfully recorded. Yet the press of other matter, and the many narrations of instances of this complaint that have been offered to the world, prevent us from attempting much more than allusion to them. That allusion will be brief.

The first case that occurred was that of a man, aged 47, who had been bitten in the left hand by a strange cat, five weeks previously to his admission into the hospital. Some spirits and some salt were the chief applications made use of, and he never was free from pain in the wound. The symptoms had commenced with malaise and general indisposition, on the 18th October. On the morning of the 21st, he was unable to put the cup to his mouth at breakfast; and at half-past 2, p.m. he was received into the institution. The spasms characteristic of this complaint were induced by the attempt to swallow water, and by the impression of air upon the skin; he was extremely sensitive to cold. Strychnia and mustard sinapisms to the stomach and spine were the means at first resorted to. He continued to get worse till near 6, a.m. of the 22d, when an enema, consisting of five grains of tobacco and a quarter of a pint of water, was thrown into the rectum. Tranquillity almost immediately succeeded; but, at 8, a.m. the paroxysms recurred, and displayed no considerable intermission subsequently. He expired rather suddenly, at a quarter past five, p.m. On one occasion, he was blown on with extreme gentleness by the physician, who stood upwards of four feet from him; he threw himself to the opposite side of the bed, averting his head, and declaring that the wind would move a 74-gun ship! The following were the main particulars elicited upon dissection.

The muscles were of crimson colour, and surcharged with blood. The latter was generally uncoagulated.

In the situation of the bite of the cat, a point of discoloration, resembling the ecchymosis resulting from a leech-bite, was observed under the skin of the thumb of the left hand. A small nervous twig was thought to be greatly reddened.

There was some effusion between the cerebral arachnoid and pia mater, conjoined with a degree of opacity of the former membrane. The bloody points in the brain were very numerous; about 3½ ozs. of fluid were contained in the lateral ventricles; the membranes at the base of the brain, of the pons, and of the medulla oblongata, were of bright red colour, injected, and adherent to the parts in question; and the membranes investing the origins of the 8th and 9th nerves were gorged with blood.

There was nothing remarkable in the spinal cord, nor in its membranes.

The upper part of the pharynx was slightly inflamed. The œsophagus presented some enlargement of its small glandules.

The cardiac end of the stomach was inflamed.

The trachea and bronchiæ were inflamed. The pleura contained a pint of fluid. The lungs were gorged with serum, blood, and sputa. In the central valve, at the arch of the aorta, were some depositions of bony matter.

The second case was that of a young man, who was first attacked with difficulty of breathing and soreness of the throat on the 18th of November. The disease was not suspected till the 20th, on the evening of which he was taken to the hospital. The case was seen in the first instance by Mr. Griffith, an intelligent surgeon of Pimlico. At 11, a.m. of the 21st, he died. A circumstantial and interesting account of the symptoms is presented. On this we cannot venture, but, perhaps, we may observe, that the sensibility to the impression of air upon the skin was more decided than even the horror of fluids. The treatment essentially consisted of the employment of the tobacco enema. It appeared that the patient had been bitten in the hand by a dog, who displayed the early symptoms of rabies, rather less than two months before he himself fell a victim to that cruel malady.

Dissection disclosed little morbid alteration, and none of a satisfactory description. The brain was not vascular, but the pia mater investing the pons varolii and medulla oblongata was injected with arterial blood, and adhered strongly to the parts beneath. The penis was, before and after death, in a state of semi-priapism.

We shall select a few remarks from those addressed by Mr. Pettigrew to the pupils of the hospital. The first refers to the period of time at which the disease most usually appears.

"George Grindley (the second patient) it seems must have been bitten between the 21st and 28th September, and his hydrophobic symptoms commenced on the 18th of November, in this respect corresponding with the period at which most commonly the disease occurs after the bite. In the majority of cases, the disease manifests itself within two months. Dr. Hamilton constructed a table (which I am at present engaged in extending, and the result of which I shall lay before you at a future time), from which it appears that the disease seldom appears before the 19th day or after the 18th month. In one hundred and thirty one cases the number from the 18th to the 30th day are only seventeen ; from the 30th to the 59th, sixty-three ; from 2 to 3 months, twenty-three ; from 3 to 4 months, nine ; 5 months, two ; 5 months and 11 days, one ; 6 months, one ; 7 months, one ; 8 months, two ; between 8 and 9 months, one ; 9 months, two ; 11 months, one ; 14 months, one ; 18 months, two ; 19 months, one ; which perhaps is, of well authenticated cases, the longest period known. All stated to have occurred beyond this time are probably fabulous, or not depending upon the bite to which they have been referred. The ancients generally ascribed forty days as the time at which the disease would become apparent, and subsequent observation has confirmed the remark. It is not improbable but that the disease may be hastened in its progress by exposure to the cold or other causes ; it however appears to be necessary that the poison should lie dormant a certain time to be fitted to exercise its virulent effects, and there are instances on record by a celebrated Italian Physician, Dr. Cocchi, in which the Small Pox has been known to occur and to go through its progress in persons who had been bitten, and who have afterwards fallen victims to the disease."

The latter circumstance is certainly remarkable, yet a little consideration may tend, perhaps, to diminish our surprise. Two diseases seldom run their course together, but two specific poisons may exist at the same time in the system. Thus, a patient with syphilis may take and go through small-pox ; measles, and scarlet fever are at times coincident ; and other similar instances might be adduced. Each specific poison has peculiar laws, and the pre-occupation of the system with one disease is evidently insufficient to prevent, although, perhaps, it may *tend* to do so, the reception of another.

Mr. Pettigrew notices a vulgar error —that dogs have really a hydrophobia, a dread of liquid. In most rabid animals there is an urgent thirst; in man alone is the repugnance to deglutition very marked or very constant. In him, it is probably dependent on the same cause which exalts the sensibility of the surface of the body, and, indeed, of all the organs of sensation. Who has not remarked the quick ear and the hurried glance of the hydrophobic patient. He is startled by a sound, convulsed by a bright object or a breath, precipitated into a paroxysm by the bare idea of what may cause one. It has been proposed, and apparently absurdly, to perform tracheotomy for this disease. The ingenious conception was founded on an imperfect acquaintance with the disease. For difficulty of deglutition is a symptom only, sometimes prominent, sometimes inconsiderable, and, perhaps, not so constant as the morbid sensibility of the surface of the body.

"Various organs of sense (says Mr. P.) have appeared to be rendered more exquisitely sensible under this disease in different cases. The intolerance of light has been noticed by several authors, particularly by Dr. Mead, who mentions the case of a girl who could not endure the light even when her eyelids were closed, and was, therefore obliged to lie with her head under the bed-clothes. The uneasiness created upon beholding mirrors, and shining substances, are of too common occurrence to need further reference on this subject. The most striking symptom in Grindley's case was his extreme sensibility to air. The sense of feeling was here the organ affected, and not a breath of air, however minute, however delicate, when directed upon him, but threw him into convulsions. It brought to my mind a case I saw several years since with my late friend Dr. Powell, at St. Bartholomew's Hospital, in which this same exquisite sensibility existed and in so high a degree, that the mere transit of a fly across the face, without coming into contact with the skin, was a sufficient cause for producing a paroxysm."

Mr. Pettigrew does not despair, nor do we, of a remedy being discovered, at some future time, for hydrophobia. In all diseases, where the lesions of organs are not very serious, it is possible that chance, or ingenious reasoning, or the progress of science, may furnish us with medicines capable of overcoming the morbid action. There was a time when mercury was not exhibited for lues, sulphur for the itch, arsenic or bark for ague. Who shall limit the advance of art?

It is much to be regretted, that we usually are called to treat hydrophobic cases when the malady has already made some progress. The incipient symptoms are obscure; they are those of some ordinary and unimportant ailment. It is not impossible, though, perhaps, not likely, that persons do recover from *such* symptoms. Be this, however, as it may, the tobacco, in the present cases, and probably other remedies in other cases, have been given at a time when the disease had made some head—a time when the experience of centuries whispers that no hope remains.

Mr. Pettigrew, like all judicious surgeons, dwells strongly on the paramount importance of preventive measures. Of these, excision is incomparably the best, and should never be neglected, where circumstances will admit of it. Two persons were bitten by a rabid dog. The wound in one was cauterized by a surgeon, near the Regent's Park. We excised the bitten part in the other. The patient who was cauterized died of hydrophobia; whilst he who had the part removed by the knife is still alive and well.

We shall conclude with Mr. Pettigrew's conclusion, expressing, at the same time, our high opinion of his accomplishments, as a surgeon and a gentleman.

"The human species is, fortunately, less susceptible of infection from the rabid poison than the brute creation. We have the authority of the celebrated John Hunter, an observer of the animal economy in health and disease, whose accuracy no one will venture to question, that a rabid dog bit four dogs and twelve human beings, that of these all the dogs died rabid and the human species escaped the infection. It is desirable, however, that excision should never be neglected, and the sooner it is performed the better; though I believe that security will be afforded by the removal of the bitten parts at any period between the infliction of the bite and the occurrence of the first symptoms of hydrophobia. A case in point upon this matter related to me by my friend Mr. Saumarez, who formerly practised very extensively in the neighbourhood of London, is of exceeding interest and importance, and may serve to give consolation to many labouring under an apprehension as to this disease. A grandmother, mother, and three children, were lying in bed, covered with but a small quantity of clothing, when a strange dog entered the bedroom and wounded the whole of them. The animal escaped, and no attention was paid to the circumstance until the grandmother, at about five weeks from the bite, became hydrophobic and died. Mr. Saumarez made enquiry into the case, and learning the particulars of the injury to the mother and three children, recommended that the parts at which the bites had been made should be excised; to this the sufferers readily assented, and the marks were sufficiently obvious to direct Mr. Saumarez in his operations. They all escaped the disease, and at the time Mr. Saumarez communicated to me this interesting case, two years had elapsed, and they were all living."

---

A Proposal, by Dr. Stewart Thorburn, of Liverpool, to dislocate the Head of the Femur from out the Acetabulum, as an important Mean towards the successful Treatment of Morbus Coxarius, or "Hip-Joint Disease."

We have received the following letter from an esteemed correspondent, Dr. Thorburn, of Liverpool. We do not entertain the views which he defends, but his proposal may safely be left to the reason and experience of surgeons. Without attempting to open up the argument, we publish the letter as transmitted to us by its author.

"For some time past, I have been intending to submit for the consideration of the profession a measure which, meanwhile, I consider will prove of great advantage, if not in all, yet in a majority of cases of *hip-joint disease.* But, on the present occasion, I shall only enter cursorily on the grounds, agreeably to which I conceive myself authorized in seriously suggesting the prudent adoption of the measure which I have to propose, and which, perchance, may startle many, viz. to dislocate the head of the femur from out the cotyloid cavity.

I would humbly beg to range myself on the side of those pathologists, who believe that, in the majority of cases, the diseased action constituting morbus coxarius essentially commences—neither in the cartilages nor synovial membrane—but in the *bones* which enter into the formation of the hip-joint. I am perfectly aware this is directly opposed to the recorded opinions of a very high authority—Sir B. Brodie; but I think the tendency of probabilities is against his, and greatly in favour of the views of those who subscribe to the belief, that the *fons et origo mali*, in hip-joint diseases, originates in the *osseous* tissue. Professor Syme observes—'it is evidently of much consequence to ascertain the nature and most efficient treatment of this disease. As an opportunity of dissecting the parts, in the first and second stages of the morbid process, very seldom occurs, being confined to those cases in which the patient dies of some other disease—the origin of the evil is still involved in considerable obscurity. Different authorities accordingly refer it to thickening of the synovial membrane, ulceration of the cartilages, and suppurations of the bones. But, though the second of these opinions be the one generally received in this country, there seems good reason for considering the one last mentioned as nearer the truth. The facts that have been collected by actual examination are in favour of this view, and the symptoms observed externally all lead to the same conclusion.

The long existing pain at *distant* parts of the limb, before any trace of disease at the part really affected can be observed, is strongly characteristic of chronic inflammation in the osseous tissue. The freedom of motion without any crepitus, that continues during the second stage, is hardly reconcilable either with ulceration of the cartilage or thickening of the synovial membrane; and the dissections that have been recorded, in which the bones were always found principally affected, afford a strong proof that they are the original seat of the malady. In the third stage there is unfortunately no want of opportunity for investigation by the knife; but then, as always happens in diseases of the joints which have advanced to suppuration, the whole articular apparatus is so involved in the destructive process, that the part primarily affected cannot be recognized. In three cases which I have dissected, (Professor SYME is speaking of his dissections up to the year 1831) at the beginning of the third stage, that is, after suppuration, but before the matter was discharged externally, the articular cartilage was sound everywhere, both on the head of the femur and on the acetabulum, except a small portion not so large as a sixpence at the centre of this cavity, where it was removed, and allowed a probe to pass into or rather through the bone. In one of these cases the synovial membrane was gelatinous, but not to any considerable extent.

The disease may then be regarded as consisting primarily and essentially in chronic inflammation of the bones composing the joint, of which the pelvic portion usually suffers alone, or at all events much more than the femur, and the practice proper for subduing it, is consequently that which has been found most efficacious in the treatment of such affections of the articular apparatus. This is counter-irritation,' &c. &c.—*See* SYME's ***Principles of Surgery***, vol. 1, p. 322.

Mr. FORD, in his ***Observations on the Diseases of the Hip-Joint***, so long ago as 1794, says, 'in every case of disease of the hip-joint which has terminated fatally, I have remarked, that the *os innominatum* has been affected by the caries in a more extensive degree than the *thigh-bone* itself,' &c.

I have adverted to these opinions forasmuch as, on a little reflection, it will be patent to the understanding of all who may peruse these observations, that the validity of, or *faith* reposed in the respective opinions of those who oppose or agree with Sir B. BRODIE's views, will materially and naturally influence the *prima facie* reception, which my proposal may meet with. After reading the analytical digest of Sir Benjamin Brodie's improved edition of his standard work on the Joints, in Numbers 41 and 42, for *July* and *October* 1834, of the MEDICO-CHIRURGICAL REVIEW, embracing (as such digest does) the most prominent views and practical points in the work at large,* I cannot find any remarks which would legitimately appear to militate against the advantages likely to emanate from the prudent adoption of the mean I propose, which, as has been said is—*to dislocate the head of the femur from out the cotyloid cavity* as early as possible after the torturing and usually headstrong disease shall have pronounced itself, through the language of its symptoms, which is neither easily overlooked nor apt to be mistaken by those who are alone entitled to public confidence by being well grounded in the principles and practice of the medical profession.

Sir B. BRODIE in common with prior and subsequent observers has noticed that the os femoris cannot be moved about without subjecting the patient to immense torture; that immediately after the complaint is detected, the hip-joint is found to be tender *whenever* PRESSURE is made on it *either before or behind*;—and admits that wherever the pain is situated, it is rendered agonizingly intense by MOTION of the *joint*. Although Sir Benjamin adds—*especially* by whatever occasions PRESSURE of the *ulcerated* CARTILAGINOUS *surfaces*

* Also of Mr. WICKHAM's,—See No. 41, from pages 68 to 84.

*against each other*, &c. yet I apprehend the pain is rendered intense by MOTION of the joint *long before the* CARTILAGES *are implicated*—before the morbid action has got the length of *ulcerating* them. The pain appears to me owing to the globular head of the femur rubbing against the other bones forming the joint, when one or other, or all of them as the case may be, are in a state of diseased action. Ought not the tendency of concurrent probabilities to incline us to attribute such friction or pressure upon, and consequent irritation of a surface or surfaces in a state of morbid action to be the immediate cause which gives rise to the *mental* sensation PAIN, referable to the hip-joint as its *physical* locality? But farther: should they not also be considered as suggesting to practical men as the grand mean (by which to be enabled efficiently to fulfil the *secondary* yet important indications on which I need not enlarge)—to *prevent the head of the femur* fretting or being fretted by FRICTION OR PRESSURE *unavoidably consequent on* MOTION *in the affected joint?* The more or less absolute the state of rest is, the more or less is the relative immunity from pain; and the longer is the disease kept in check, and the recovery by nature's efforts promoted. Let any man observe in his own person or in that of another, how the natural action of the lungs during RESPIRATION perceptibly and notably moves the hip-joint, and he will be constrained to admit, from this alone, that rest cannot ex natura rerum absolutely prevent the hard parts grating against each other, to prevent which, unquestionably constitutes *the* great and one thing needful in the scientific and efficient treatment or morbus coxarius. Would not the measure which I have ventured to propose effectually remove, not only the cause of pain, which plays a most important part in the tragedy of this disease, but the same cause whose continued operation irritates, as I beg leave to think, a nasty inveterate morbid action—call it chronic inflammation, or what you will—into full development; and which by extending to, ultimately implicates the cartilages and ligaments of the articulation, to the destruction partial or total of its organization? What is the consequence of this disease allowed to run its course, or when unrestrained by surgical interference? Is it not as nearly as may be as I now delineate? The disease we know may run its course in a period varying from a few months to as many years. The analysis of outward *symptoms* not only renders it possible, but their conversion into SIGNS highly probable, that the action extends *from* the OSSEOUS TISSUE *to* the synovial membrane, interarticular, cotyloid, and capsular ligaments, which are destroyed by "ulcerative absorption." Nor is this all: emaciation of, and abeyance of the muscular tonicity in the soft parts around the diseased locality, have *simultaneously* been in progression; and a natural and important link in the complicated chain of cause and effect, namely, "spontaneous" dislocation of the joint, as it has figuratively but most erroneously been styled, takes place. And what most interesting phenomena do not many, indeed all of *such*, cases present to the notice of nature's secretary, the philosophic observer! Is there not a perceptible check to the progress of the disease—a breathing time as it were, even in cases apparently past hope? and in those less complicated, is there not an immediate cessation of pain for which the wretched sufferer pours forth a prayer of thanks, and communicates unasked intelligence of the fact of his relief, to the attendants? Have not cases again and again been completely arrested in their otherwise fatal career, when such dislocation occurred, though tardily brought about by the very disease itself? The nearest approximation to absolute rest in such cases can be secured, and other means superadded and successfully too, in suspending the disease through medium of *anchylosis*, or the formation of a false but *useful* joint, with a more or less perfect recovery of the general health.

To my mind an essential feature in such recoveries is, the globular head of the femur *leaving the cotyloid* cavity. To such, in great measure, if not entirely, must be attributed the subsidence of the disease, and the steady success, transient or permanent, of subsidiary means of cure, before the development of abscesses, burrowing fistulous sores, and severe constitutional disturbance have exhausted the renitent staminal powers of the organism. In conclusion; for the present suffice that I say, it has been from dwelling and reflecting on considerations akin to those, merely outlined as it were here, after analyzing as correctly as I could, the sequence of effects from causes in their most probable order of dependence, that I have been induced to submit to the consideration of such practical men as are guided by observation and enlightened by the revealing torch of pathology, these queries—

1st. Would not the artificial dislocation of the head of the femur from out the cotyloid cavity, in a majority if not in all cases of MORBUS COXARIUS be among the most expedient measures which in the present state of surgery could be adopted in the treatment of this disease?

2nd. If so—ought not the mean proposed to be resorted to as early as possible, (or when?) after the pronounced PRESENCE of the disease?

I am, Gentlemen, faithfully yours,

J. STEWART THORBURN, M.D,

*Liverpool, Oct. 30th,* 1834.

P.S. Having exhausted my paper, I can only allude to the interesting but peculiar views ascribed to *Monsieur Dzondi,* who is alleged to speak upon the strength of thirty years' experience. But surely it must be of the exception, not the rule, when he is represented from the *Algemeine Medic. Zeitung* as asserting that, "the seat of the inflammation at first is always exterior to the joint; it is neither in the bone itself, in the synovial membrane, fibro-cartilage, nor in the synovial glands, but invariably, according to the experience of M.D. is in the outer surface of the articular capsule, in the fibrous structures about it, and in the periosteum of the bone round the circumference of the acetabulum, and of the upper part of the femur itself. The limits within which the inflammatory process is usually at first circumscribed, are about one inch in extent all round the rim, or edge of the acetabular cavity; but the whole of this space is not generally affected simultaneously; the irritation may be concentrated either at one part of the capsule, or of the periosteum of the os ilii, or of the cervix femoris, or round the border of the acetabulum, or on the great trochanter."—*Vide Medico-Chirurgical Review,* Number 41, p. 239.

---

### PENDULOUS TUMOR GROWING FROM THE EXTERNAL EAR.*

It appears that goitre prevails in the valley of Nipal, in Hindostan, and that a pendulous tumor growing from the external ear is also frequent in the natives of that district. The two morbid growths are often observed in the same individual. Dr. Campbell presented to the Medical and Physical Society of Calcutta, two tumors which he removed from the ears of a female. They grew from the helices and drew down and doubled the ear over the external meatus auditorius, so as greatly to obstruct hearing. The surfaces of the tumors were uneven, and their feel fleshy, but firm; their internal structure is described as resembling that of mammary sarcoma. They were removed by simple incision with a scalpel, and weighed together 24 ounces. The wounds soon healed, and the ears resumed their natural position. The patient from whom these tumors were taken, is affected with a large bronchocele; her eldest daughter, aged nine years, has a tumor, the size of a walnut, attached to each external ear, and another daughter, aged six years, has a goitre of three years growth, which is as large as an orange. In the same communication the author states, that bronchocele very often occurs in animals in Nipal, lambs and kids being often born with remarkable morbid development of the thyroid gland.

* Trans. of the Med. and Phys. Society of Calcutta, Vol. VI, 1833.

Occlusion of the Ductus Communis Choledochus.*

An European pensioner at Chunar, æt. 31, who had been for eleven years a resident in India, suffered for three months from symptoms of chronic dysentery, attended with pain in the region of the liver; for which he was repeatedly salivated, but without benefit; he gradually sunk under the dysenteric symptoms. On post-mortem examination, the mucous membrane of the great intestines was found in a state of ulceration; the liver was of a dark color, but not otherwise diseased; the gall-bladder contained a small quantity of dark viscid bile: the Ductus Communis Cholidochus was compressed by an enlarged gland, seated in the capsule of Glisson, and its canal was obliterated. The author refers to the cases of occlusion of the biliary ducts, recorded by Mr. Twining, in the 5th vol. of this Society's Transactions, in proof of the frequency of obliteration of these ducts; and also mentions Portal's case of obliteration of the biliary ducts as well as the gall-bladder, related in the treatise *Sur la nature et le traitement des maladies du foi;* Dr. Farre's case in his *Morb. Anat. of the Liver;* Dr. Todd's case in the 1st vol. Dublin Hospital Reports, and Dr. Percival's case in the 2nd vol. of the same work.

---

Operation of Lithotomy and Prevalence of Calculous Complaints in India.

It appears to be now an established fact, that calculous complaints are frequent among the native population of India. In the last volume of the Calcutta Society's Transactions, are several communications on this subject. One gentleman, Mr. F. H. Brett, of Shajehanpore, relates the particulars of twenty-two cases in which he performed the lateral operation of lithotomy. The cases themselves present no peculiar features of importance. Mr. Brett observes:—

"I have been unfortunate in losing four patients out of 22, but I have never rejected any that have presented themselves, where there was a possibility of relieving human suffering of so intense a character. Three of the fatal cases did not request my aid until their constitutions were completely exhausted by the protracted irritation. The unfortunate terminations (excepting the cachectic child No. 13), happened likewise in the hot season, when the weather was unusually sultry and unhealthy. Two of these were carried off by Tetanus, and one of the four fatal cases survived the operation a month, having been a martyr to the disease, *thirty years*.

In the last fifteen operations I employed merely a common scalpel, having lost the only beaked knife in my possession; and finding this single instrument simplifies the operation, I now generally prefer it to any other, having required no other instrument than the scalpel where the calculi were large, as in cases 15, 17, and 20."

He always employs the straight staff, and he thinks that one great advantage arising from it, is its power of lowering the prostate gland and neck of the bladder towards the perinæum, when its handle is only slightly inclined towards the operator. The latter is not obliged to cut so deep as when he uses the curved staff, the prostate being very distinctly "seen" on completing the second incision. On glancing at the cases we are struck by the prevalence of oxalate of lime as an ingredient in the calculi.

Mr. Burnard, of Benares, relates five cases in which he performed the operation. The patients all did well. Mr. Darby, of Almorah, details four, in which the operation was equally successful. The latter surgeon used a common scalpel, a grooved staff, and forceps. Mr. Bell, of Hawalbang, presents three cases. He states that the native surgeons sometimes cut for the stone much after the old method "on the gripe" as it was termed, but they will never undertake the operation un-

* Ibid.

less an application has been first made to the civil power, stating, that in the event of death, the operator is not to be prosecuted by the relatives of the patient. A prosecution of this nature did actually take place some years ago at Sireenuggur. A similar state of things exists in Turkey, and has characterized the barbarous periods of most nations.

It would seem that the Indians bear the operation of lithotomy well; for, the surgeons are miserably stocked with tools, and operate under great consequent disadvantages. Mr. Bell, for instance, was compelled to employ a common country blacksmith to construct his instruments, and in one of his cases the forceps broke, and a stone was in consequence left in the bladder.

Dr. M'Gregor, of Loodianah, communicates another case in which lithotomy was successfully performed. He employed the straight staff. With a notice of a case of sloughing of the scrotum, in consequence of the impaction of an urinary calculus in the urethra, we dismiss the present subject.

A sepoy was received into the hospital at Subathoo, in May, 1832, with much swelling and sloughing of the scrotum, and great fulness and tenderness in the perinæum.

"He says the swelling only commenced nine days ago, although for the last 11 years he has experienced the greatest difficulty in passing urine, in consequence of a hard substance lodged in the urethra; this was first felt in the perinæum 11 years ago, since which time it has slowly, by the efforts of the bladder and assistance of his fingers, been brought to within an inch of the orifice of the urethra, where it has been fixed for the last three years. At first the bladder was emptied with much pain, the urine flowing guttatim; latterly, from having squeezed the parts frequently in his fingers, the difficulty became less. On examining the parts, I found the prepuce covering the glans penis, and with an opening barely sufficient to admit the small end of a probe; the hardness he mentioned was easily felt where the urethra joins the glans, and evidently caused by a small calculus; he was not aware of any increased difficulty in making water just before the swelling appeared, but first felt the uneasiness about the perinæum, after a long morning drill, nine days ago. The prepuce was divided with a scalpel, and the glans was uncovered; the stone was then easily taken out by enlarging the orifice of the urethra: about one half of the stone was lodged in the substance of the glans, and from its form (mulberry), it came away in small grains; the portion projecting into the urethra came away entire, and is forwarded with this memorandum. A catheter was introduced after this, and the bladder emptied without any difficulty. I then made an opening into the tumor in the perinæum, and discharged about 1½oz. of fœtid watery matter, without relieving in any way the tension of the scrotum. On scarifying the lower part of it with a lancet, a watery discharge came away; the catheter was withdrawn, and a large warm poultice put over the whole."

It is sufficient to observe in continuation, that the entire scrotum sloughed, that granulations arose on the exposed testicles, that the latter became gradually encased in skin, and that Mr. Mitchelson, the surgeon and reporter, indulges the expectation of the ultimate accomplishment of a respectable sort of scrotum.

We lately saw an instance of sloughing of the scrotum from effusion of urine, the latter being occasioned by a small calculus obstructing the urethra. The result was less fortunate than in the case detailed above.

In conclusion we will offer but one remark. The advance of information on the subject of urinary maladies demands, on the part of the narrator, an acquaintance with, and record of, the state of the urinary secretion. Without such, the knowledge of the case must be imperfect; the account of it comparatively valueless.

Yet we find we must indulge in some further "novissima verba." Lithotrity has been introduced and successfully practised in Calcutta. Dr. Cassanova presents the particulars of two cases in which he adopted the method of Civiale with success. He does not appear to be aware of the improvements effected in the operation by the use of the "percuteur." The hammer or the screw will ultimately supersede the drill-bow and the saw.

---

### Dr. Edwards on the Physiological Characters of the Races of Mankind.

M. Amédée Thierry has published a History of the Gauls, which gave rise to a letter, and to many speculations, on the part of no mean physiologist, Dr. Milne Edwards. For what we know of either, we are indebted to the Phrenological Journal, for December, 1834. We shall not attempt a consistent account of all that Dr. Edwards thinks or has observed. The gist of his letter is an attempt to prove, that certain types prevail in modern nations, by which we may recognize their forefathers and origin. He starts with combating the popular belief, that tribes and nations have become extinct. He argues with much force in the following strain:—

"When a people is conquered, and has lost its independence, as it no longer forms a nation, it ceases to exist in history; and we are tempted to believe that in such revolutions each disaster annihilates the previously existing races. But an attentive study of languages enables us to detect, in those spoken at the present day, the ancient idioms which have formed them, and thus to trace, in countries where otherwise we should never have suspected it, an uninterrupted connexion between the ancient and modern inhabitants. If, then, the forms of speech leave traces which betray their ancient origin, what are we to think of the physical characters of the race? Are they less permanent? Do we retain nothing of the features of our ancestors? Has climate so changed them that they can no longer be recognized? Has the mixture of races confounded every thing? Has civilization regenerated every thing? Has decay degraded every thing? Has force exterminated or expelled entire people? Such are the questions which must be briefly examined, before coming to the observations which are the subject of this work."

We agree in the main with Dr. Edwards. Yet we would not wish to depend more upon his line of reasoning than it will safely bear. A language may subsist when its original proprietors are gone. The admixture of Greek roots, in the modern European tongues, is out of all proportion to the extant descendants of the old Greek race; indeed, both Greek and Latin words have been plentifully soldered into our own language in the last century. Dr. Johnson, it is well known, made almost exclusive use of classical derivatives, and added many to the then existing stock. The writer of the Letters of Junius, on the contrary, has preferred the Saxon portion of our tongue, and those Letters constitute a noble specimen of its terse and manly vigour. It is easy to imagine that a race may gradually become extinct, yet survive long enough to impress its language and customs on its successors. To revert to Dr. Edwards.

In order to demonstrate his proposition—that the types of former races still remain—Dr. Edwards proceeds to clear away the arguments which the influence of climate and the mixture of races might afford.

He denies, or at least disputes, the power of climate to effect material alterations, and he instances the Jews, who appear to preserve, in Asia and in Europe, those distinctive features depicted, 3000 years ago, in paintings in the tombs of Egyptian Kings.

The mixture of races presents a more complex difficulty to unravel. Yet Dr. Edwards combines the process of cutting and untying, to disentangle the unyielding knot.

"First (he proceeds), we must consider the relative number of the two races. Supposing *a very great disproportion*, the type of the smaller number will finally disappear. If a Negro and a white produce a mulatto, this mulatto with a white produces an individual nearer to the white; and after five and sometimes even four crossings with white blood, the black taint can no longer be perceived. The same is observed in domesticated animals. This conclusion, at first, appears unfavourable to the search after ancient races among modern nations; and it would be so in the case of such races as had formed but a minute fraction of the mass; but where the mass has been great and preponderating, this principle shews, on the contrary, that the type of the race must still exist. If, then, where no restrictions as to mixture of races exist, the least numerous, if the disproportion be great, finally disappears, still less will the type of the more numerous be altered, if, as in most cases occurs, such restrictions do exist.

Let us now take the other extreme case, namely, where the two races are *equal in number*. What is required, that both should disappear, and form only one intermediate type?

Each individual of the one race must unite with an individual of the other, or at least each race must have nearly an equal share in the amalgamation of physical characters. Such are the conditions absolutely necessary; and if their occurrence be not impossible, it is, at least, in the highest degree improbable.

When animals of different species are crossed, they produce an animal of an intermediate type, or a mule; but when different varieties of the same species are mixed, the result is often quite different. M. Coladon, of Geneva, made a very striking experiment, which bears strongly on this point. He procured a great number of white mice, as well as of common brown mice, studied their habits, and found means to cause them to breed. In his experiments, he always put together mice of different colours, expecting a mixed race; but this did not occur in one instance, All the young mice were either white or brown, but each type was produced always in a state of purity.

Even in the case of varieties of the same species, we have an intermediate type or mule, but this is when the varieties differ most from each other: when, as in the case of the mice, they approach very nearly, mules are not produced. In both cases we see one common principle, namely, that the mother often produces a being of a type different from her own—less so, however, in the latter case. The same principle is seen even in the same variety; for here also the mother, in producing a male, gives birth to a being whose type differs, and in some cases differs very much, from her own.

Now, the same is observed in man. The varieties which differs most strongly, such as the Negro and white, when crossed, produce mules; and when varieties more nearly resembling each other are crossed, the descendants sometimes resemble one parent, sometimes the other, sometimes both. This is the cause of the great variety observable in modern nations; among which, however, we can always observe specimens of the pure types which have entered into their composition. Thus, even if two races having considerable resemblance to each other, and in equal numbers, were to mix without limitation, the original types would still frequently occur in their descendants.

Another cause which prevents the disappearance of the original types, where there has been no great disproportion of numbers, is the geographical distribution of the races. They cannot be so thoroughly mixed that the one or the other shall not predominate in some district, where, of course, the type of the race so predominating must exist.

A type may occasionally disappear by extermination. Thus the Guanches, savages who inhabited the Canary Isles, have disappeared; but their number was small, and they were confined to small islands. The Caribs, likewise, for the same reason, have almost disappeared from the Caribbee Islands, although they are said still to exist on the continent. But it is impossible to extirpate a numerous nation, more especially when they have attained a certain degree of civilization. In that case, it becomes the interest of the conquerors to preserve the conquered people as slaves, and not to destroy them; and we have no example in history of a *whole people* sacrificing themselves rather than submit to such slavery. On the other hand, we must suppose an incredible rage and cruelty on the part of the conquerors, if a whole people is to be exterminated. When it was proposed to Genghis Khan, by some of his counsellors, to extirpate the Chinese whom he had conquered in the north of China, as being useless to the conquerors, one of his ministers, Yeliu-thou-tsai, made the emperor observe, that in advancing towards the south, his armies would be in want of many things which it would be easy to procure by imposing on the conquered people contributions, not oppressive, of money and provisions.—How, then, could it be said that such a people was useless to the state? This reasoning prevailed, although the cruelty of the Mongols was atrocious; and such reasons will always oppose the extermination of populous nations, possessed of some civilization.

A nation, that is, a numerous people, may be dispossessed of a large territory. This, however, has rarely happened, and only in the case of savages. It has occurred in America, but not in Hindostan. Where industry exists, the chiefs cannot induce a nation to emigrate in a body; and if conquered by a new tribe, the latter expels a portion to obtain room, if nomadic, but preserves the rest, as slaves, as auxiliaries, or as tributaries. These conclusions are confirmed by history; and M. Abel Remusat has even been able, by comparing language with history, to discover nearly all the nomadic tribes of Asia in their primitive seats, notwithstanding the numerous revolutions and conquests which have occurred in that quarter of the globe."

In applying these principles to practice, Dr. Edwards remarks that, in the nation which we study, we must notice if one or more types exist, and then endeavour to trace them to their origin. The characters which most strongly distinguish a type, are those which are drawn from the proportions of the head and features. A type cannot be correctly ascertained from the examination of an individual. To be satisfactory, many must evince it.

It appears that the greater part of Gaul was occupied by two great families, differing in social state, in language, and in habits—the Gauls and Cimbri. The former were the ancient inhabitants of the country, and are probably most numerous. M. Thierry has placed them in the south-east of France. The Cimbri, the conquerors, and probably inferior in respect to numbers, are chiefly placed by the same author in the north of France, in the Belgium of Cæsar, and in Armorica. Such are the results of the investigations of the historian. Let us turn upon them the observations of the physiologist.

"In travelling through France, Italy, and a part of Switzerland," we are employing the analysis of our phrenological contemporary, "Dr. E. had scarcely reached the frontiers of Burgundy, when he began to observe a union of features which constituted a particular type. This became more marked and frequent as he penetrated into the country, especially from Auxerre to Châlons. He arrived in this latter town on a market-day, and immediately repaired to the market to study the faces of the peasantry from the surrounding country. He was astonished to find a great many of them totally different from those he had first observed, and forming a strong contrast to them. During the rest of his journey in Burgundy, the first type occurred frequently, and continued in the Lyonnais, in Dauphiné, and in Savoy, as far as Mont Cenis. There were in this large district many shades of colour; but, with the exception of the group at Châlons, only one well-marked type of head and face."

That type is as follows:—The head is nearly spherical. The forehead of middling size, somewhat arched, and retreating towards the temples. The eyes are large and open. The nose is nearly straight, and rounded at the point. The chin is likewise round; and the stature is middling. In a word, the head is more round than oval, the features rounded, and the stature middling. This type occurs then in the East and the South-east of France, where M. Thierry, as we have already stated, has placed the original Gauls.

In Tuscany Dr. Edwards found another type, represented by the busts and the pictures of Dante. The physiognomy of that noble poet is peculiar. The head is long, not broad; the forehead high and well-developed; the nose curved, so that the point of it droops whilst its wings are raised; and the chin is prominent.

"Dr. E. saw at Radicofani people who possessed this type, and one of whom was the image of Dante. He had also observed it in the busts of many of the Medici, and other distinguished men of the Republic of Florence; and even traced it in some Etruscan bas-reliefs. He continued to observe it at Bologna, Ferrara, Padua, and the intermediate towns. It was very frequent at Venice. When examining at this last place the picture of a saint painted by one of the Venetian school, the cicerone desired him to observe how much it resembled Dante. In the Ducal Palace he observed that a great majority of the Doges, whose portraits he saw, had the same character.

In proceeding towards Milan, this type became still more frequent, and was sometimes absolutely caricatured. In one village where he stopped for an hour or two, he saw a number of peasants, and could scarcely take his eyes off them, so great was their similarity to those whom he had seen in the market-place at Châlons. Being now in Cisalpine, as he had formerly been in Transalpine Gaul, he naturally concluded that this was a Gaulish type. In crossing the Alps, he met first with a German type, then with a Burgundian, and finally near to and in Geneva, with the type observed at Châlons and in Tuscany. Here, then, was a population composed of two races, each having its own type, and forming a complete contrast to each other. The one observed in Burgundy, Dauphiny, Savoy, and the Valais, having the head more round than oval, and rounded features, with a middling stature. The other, observed in Tuscany, at Geneva, and at Châlons, having the head long, the forehead broad and high, the curved nose, the prominent chin, and a tall stature."

Our readers will suspect, what is the case, that the type thus pursued from the Somme to the Seine, at Châlons and in Tuscany, is no other than that of the victorious Cimbri.

This illustration of the views of Dr. Edwards is sufficient to awake the attention and excite the reflections of those who take an interest in these pleasing, though not conclusive inquiries. It is evident, of course, that demonstration, or even satisfactory proof, is unattainable—that much must depend upon the faith, much on the imagination of the observer—and that all which can result is a theory more or less probable, and more or less ingenious. The dissatisfied historical philosopher may hint, that either the types of nations have multiplied to more than Dr. Edwards' principles admit, or the primitive families of man were more numerous than physiology or theology allows. It is almost certain that modern Europe was peopled by migrations from the North of Asia, and even the reasoning of Dr. Edwards would oppose the propagation of many types in that single quarter. This consideration may, perhaps, be overstretched, yet *if* so many types could be generated in a comparatively limited period, in a limited space, and with a limited population, it is difficult to imagine, or, at all events, to believe, that the mixed and motley breed of Europe, the kennel of all the blood-bounds of the North, should maintain unchanged the features of its savage conquerors.

The Phrenological Editor laments that Dr. Edwards was unacquainted with *his* science, and hopes that the mere physiological observer may be aided by the more profound phrenological philosopher.

